# The impact of awareness on affective emoji priming in visual word recognition

**DOI:** 10.1038/s41598-025-34117-w

**Published:** 2026-01-05

**Authors:** Demian Stoianov, Elisabeth Beyersmann, Nenagh Kemp, Signy Wegener, Srdjan Popov

**Affiliations:** 1 International Doctorate for Experimental Approaches to Language, Universities of Potsdam, Postdam, Germany; 2 International Doctorate for Experimental Approaches to Language, University of Groningen, Groningen, The Netherlands; 3 International Doctorate for Experimental Approaches to Language, Macquarie University, Sydney, Australia; 4 International Doctorate for Experimental Approaches to Language, Newcastle University, Newcastle upon Tyne, Australia; 5 Center for Language and Cognition Groningen, University of Groningen, Groningen, UK; 6 School of Psychological Sciences, Macquarie University, Sydney, NSW Australia; 7 School of Psychological Sciences, University of Tasmania, Hobart, Australia; 8 Australian Centre for the Advancement of Literacy, Australian Catholic University, Sydney, Australia

**Keywords:** Emojis, Affective priming, Lexical decision, ERPs, Continuous flash suppression, Visual consciousness, Neuroscience, Psychology

## Abstract

Emojis have become integral to digital communication, yet their impact on downstream cognitive processing is not fully understood. In two lexical decision experiments, we examined the behavioral and electrophysiological effects of emoji primes that varied by emotional valence (positive, neutral, negative) and face-status (face vs. non-face). Experiment 1, where clearly visible primes were presented for 100 ms, revealed a positivity advantage, with positive emojis facilitating responses and modulating both early (P1) and late (LPP) ERP components, along with a response-locked positivity indicating enhanced motor preparation. Negative emojis elicited early (P1, P2) ERP modulations but did not significantly alter behavior. Face emojis also increased early perceptual components (P1, P2/P3a), suggesting that face-like properties capture attention automatically. Experiment 2 employed continuous flash suppression (CFS) to limit conscious prime perception. Under these conditions, positive emojis no longer facilitated responses, and face-status effects disappeared. Negative emojis, however, showed robust early negativities in the ERPs and a trend toward slower response times, consistent with the automatic vigilance account. Overall, the findings indicate that conscious perception is crucial for the positivity advantage of emojis to manifest, whereas negative stimuli can still engage early neural processes and inhibit performance under limited conscious processing.

**Supplementary Information:**

The online version contains supplementary material available at 10.1038/s41598-025-34117-w.

## Introduction

Emojis have become ubiquitous in computer-mediated communication for their ability to convey nonverbal cues akin to facial expressions and gestures^1^. The current study explored whether and how emojis influence written word processing. Emotional cues tend to capture attention automatically^2^ and prompt involuntary affective processing^3,4^, thereby modulating cognitive functions such as attention, perception, and semantic integration^5,6^. Despite their widespread use, little is known about how rapidly and automatically emojis are integrated during reading. This study employed EEG recordings to scrutinize the influence of emojis on visual word recognition and the continuous flash suppression (CFS) paradigm to assess this influence in situations where participants were not consciously aware of the prime.

One key approach to investigating how nonverbal emotional cues influence visual word recognition is the affective priming paradigm^7^. In this paradigm, primes and targets vary in emotional valence, creating a match (i.e., congruent condition) or mismatch (i.e., incongruent condition), and the performance difference between these conditions indexes the priming effect. To avoid confounds of semantic or associative relationships inherent in word stimuli^8^, pictorial primes are commonly used, isolating emotional influences from linguistic associations. According to a review by Rohr and Wentura^9^, facilitation in congruent pairs under these conditions may result from (a) response compatibility (i.e., when both the prime and target require the same response), (b) semantic overlap between their affective concepts, or (c) perceptual–attentional modulation by emotional content (i.e., when the emotional salience of the prime enhances early sensory processing and directs attention toward the target). Combining affective priming with a lexical decision task, in which participants discriminate words from nonwords rather than explicitly evaluating their valence, reduces the influence of response compatibility^10,11^. Thus, observed priming effects under this design must primarily reflect stimulus–stimulus (S–S) processes such as encoding facilitation^12^, affective matching^8^, conflict reduction^13,14^, or mutual facilitation^15,16^.

At the same time, findings from affective priming in lexical decision tasks often reveal valence-specific effects, with positive items showing stronger facilitation^17,18^. Recently, Stoianov et al.^18^ extended affective priming paradigms to emoji-primed lexical decision tasks, in which participants identified word targets preceded by emoji primes varying in emotional valence. Emoji primes (positive vs. negative) were paired with target words of matching or mismatching valence in an emoji–word priming lexical decision task and found that only positive primes produced robust congruency benefits. Negative primes yielded no clear facilitation, hinting at enhanced integrative processing of positive emotional cues. Supportive evidence emerged in an EEG study by Kissler and Koessler^17^, who employed images from the International Affective Picture System (IAPS) as primes and emotional words or pseudowords as targets, showing faster and more accurate responses to positive stimuli. Stimulus-locked event-related potentials (ERPs) revealed enhanced early posterior negativity (EPN) and late positive potential (LPP) for positive primes, demonstrating their ability to modulate attention and perception without substantial semantic processing. A fronto-central negativity in response-locked ERPs emerged specifically for positive pairings, suggesting early involvement of motor-related regions in response facilitation, again emphasizing a positivity-driven processing advantage. This advantage in processing positive items relative to negative or neutral ones is often referred to as positivity bias^19^ and it has been demonstrated across face, image, and word stimuli^20^. One explanatory account, the “density hypothesis,” suggests that positive information is more densely interconnected within semantic memory, resulting in richer representations and faster access^21^. In contrast, alternative perspectives emphasize the evolutionary salience and distinctiveness of negative cues^22^, which may be more multifaceted and thus harder to classify unambiguously^23^. Similarly, Santaniello et al.^24^ proposed that the recognition memory advantage for negative content could prolong attention disengagement^25,26^, potentially counteracting the speed advantage sometimes observed for positive stimuli.

Moreover, the neutral-target variant of affective priming^27^, when participants evaluate or identify neutral items while the prime conveys a clear positive or negative valence, also reveals similar valence-based priming effects^28,29^. For instance, Stoianov et al.^29^ conducted a series of four experiments, where participants were presented with positive, negative, or neutral emoji primes immediately followed by neutral word targets. In Experiments 1 and 2, the primes were clearly visible (100 ms), whereas in Experiments 3 and 4, the primes were shown for just 30 ms and sandwiched between forward and backward black-square masks. Across these experiments, positive emojis consistently facilitated word recognition, while negative emojis showed no measurable impact without masking. However, under masked conditions, there was a marginal inhibitory effect for negative primes, suggesting that negative valence can sometimes exert subtle effects when conscious awareness is minimized. These findings underscore a recurring positivity advantage and highlight the need to explore the neural correlates of such valence-specific priming for neutral targets to determine how prime valence affects lexical decision processing at different temporal stages. Current behavioral and electrophysiological evidence indicates at least two stages in shifting the target’s perceived valence: first, the prime’s emotional features must be registered^30,31^, and second, the prime-driven affect “leaks” onto the neutral target^32,33^, possibly through affective misattribution^34,35^ or resource competition^13,28,36^. An ERP approach could elucidate whether the behavioral patterns^29^ were driven by resource competition, affective misattribution, or attentional biases. Furthermore, if sandwich masking of 30 ms emojis does not completely obscure face-like features, any facial cues might still trigger early face-sensitive or emotional pathways, potentially complicating interpretations of automaticity. Overall, an ERP investigation that compares early versus late time windows can help reveal whether positivity-driven facilitation arises from low-level perceptual gating, higher-level semantic or evaluative processing, or a combination of both.

More generally, a debate persists over whether affective priming can occur subliminally (i.e., outside conscious awareness) and how successfully salient emotional picture primes can be masked to stay below the threshold of conscious detection^37,38^. Some contend that emotional primes (especially facial expressions) can bias subsequent behavior even when suppressed^39,40^, whereas others maintain that high-level details (e.g., semantic meaning, facial identity) may not survive interocular suppression^41–43^. Moreover, traditional brief masking techniques often fail to fully suppress emotionally salient face-like images^44,45^.

By contrast, Continuous Flash Suppression (CFS) can prolong invisibility for several seconds by displaying a series of dynamically high-contrast Mondrian masks to one eye while presenting the prime to the other^46^. Such extended suppression allows researchers to examine whether stimuli can be integrated into higher-level information outside awareness^47,48^. For example, several studies demonstrated privileged facial expression processing without conscious perception^49–51^. Yet others propose that such facilitation might be derived from lower-level properties like contrast or spatial frequency^52,53^. Schlossmacher et al.^54^, for example, reported no differential ERP responses to fearful or happy faces in a strict CFS condition, challenging earlier work suggesting that emotional content “breaks through” more readily. Moreover, later studies show that task demands leading to higher attentional load can restrict or nullify subliminal emotional face processing^55^.

Neuroimaging data reveal that subliminally presented emotional faces can modulate the N170 face-specific component^56^ and that affective signals may reach early visual cortex^57^ as well as subcortical structures. For example, fMRI findings have demonstrated that activity in the amygdala to suppressed negative faces covaries with superior colliculus^58^ and pulvinar^59,60^ activation. Such subcortical pathways provide a rapid but low-resolution route for detecting behaviorally salient stimuli, suggesting partial extraction of emotional value under CFS^61^. Moreover, Ludwig et al.^62^ showed that both dorsal and ventral visual streams are influenced by visibility, although the ventral stream exhibits a stronger link to conscious awareness, and Cao et al.^63^ extended these findings by demonstrating that face identity can be encoded in higher-level areas (e.g., the right fusiform face area) under shallow CFS. In line with these observations, a diffusion-model study^64^ found that facial identity and emotional content can be processed under CFS, mapping onto different model parameters (i.e., drift rate for identity, and nondecision time for valence).

Finally, converging evidence suggests that emoji processing depends on both emotional valence^18–21^ and face-status^65–67^, although their interaction under affective priming remains unclear. Face emojis can convey emotion as effectively as human faces^68^ and modulate face-sensitive ERPs^69^ (i.e., P100, N170 and LPP), albeit with generators that are partially distinct from those for real faces^67^. By contrast, non-face emojis tend to be more ambiguous^70,71^ and semantically complex^72^ than face emojis. These findings suggest that face emojis may afford faster extraction of affective value than non-face emojis; yet prior ERP priming studies^73,74^ have not dissociated valence from face-status. Furthermore, because behavioral measures can be insufficiently sensitive to subtle emoji effects^75,76^, any interaction between valence and face-status is more likely to manifest in ERP measures.

### Present study

The present EEG study investigated how the emotional valence and face-status of emoji primes influence subsequent lexical decisions on neutral words (see Supplementary Table S1 for examples). We aimed to identify ERP correlates associated with these factors by examining both stimulus- and response-locked activity. Prior work has reported emoji-related effects across numerous ERP components, including P1^77^, N1^78^, N2/P2^74^, EPN^17^, P300^28^, N400^72^, and LPP^17^. Early visual components P1 and N1 (~ 80–150 ms), which index perceptual sensitivity and attentional gating, can be modulated by salient socio-affective, face-like stimuli, including emojis^73^. The fronto-central P2/N2 complex (~ 150–350 ms) is sensitive to conflict monitoring and inhibitory control demands^79,80^, and is assumed to reflect the top-down inhibition of emotional processing for task-irrelevant cues^32^. The occipito-temporal EPN (~ 200–300 ms) indexes prioritized perceptual encoding of emotionally salient stimuli and scales with arousal^81^, while being strongly attenuated by inattention^82^. Despite temporal overlap between EPN and N2/P2, these effects are considered separable due to their distinct topographies. Later positive ERPs (~ 300–600 ms) include the P300 and LPP. Contemporary integrative accounts emphasize their close functional relation as graded reflections of stimulus significance^83,84^, linking context updating, attention, and memory with continued allocation of resources to motivationally salient content. We therefore model them jointly as a late-positivity continuum over 300–450 ms, treating effect duration as the primary dissociation. The N400 indexes semantic access and integration and is not expected to vary here because prime–target semantic congruency was not manipulated.

Hence, to assess whether priming with emojis of varying emotional valence and face-status modulates early and late ERP components linked to visual perception, attention, semantic processing, and response preparation, we conducted two experiments. Experiment 1 employed clearly visible emoji primes presented for 100 ms, thereby tapping into supraliminal (i.e., above the threshold of consciousness) processing. Experiment 2 utilized a CFS paradigm, rendering the emoji primes invisible to participants, thus explicitly targeting subliminal processing. In both experiments, we tested three planned comparisons: one for face-status (face vs. non-face) and two for valence (positive vs. neutral and negative vs. neutral). The study design, data collection, and analysis plan were preregistered at https://aspredicted.org/zs3k-kr7w.pdf.

## Experiment 1

We hypothesized that, similar to findings from human face processing^2–4,73^, face-like stimuli should elicit enhanced early perceptual processing, reflected among early components (i.e., P1, N1 or N2/P2). Findings from the neutral-target variant of affective priming^28^ have demonstrated sensitivity to the prime in both P1 and P2, although the respective contributions of valence and face-status remain unclear. Given inconsistent and limited prior evidence, we expected greater early positive activity for targets primed by face versus non-face emojis within 100–300 ms after target onset, rather than a modulation restricted to a single component. Based on previous findings^18,29^, we did not expect a face-status difference in behavioral measures.

According to evidence for a positivity advantage^17,18,29^, positive (vs. neutral) emojis were expected to modulate components related to emotional processing (EPN, LPP), attention allocation (P300), and response preparation, thereby facilitating lexical decision performance. Based on prior ERP findings^17^, this facilitation should manifest as larger amplitudes for positive than neutral primes in the EPN (200–300 ms post-target), P300 (300–400 ms), and LPP (300–450 ms) in stimulus-locked ERPs, as well as increased response-locked activity during the ~ 400 ms preceding the response. Consistent with integrative accounts linking attentional and evaluative processing with stimulus significance as a continuum, we treat P300 and LPP as a late-positivity continuum over 300–450 ms, considering effect duration as the key dissociation.

In line with prior affective-priming findings^18,22,29,85^, negative (vs. neutral) primes were expected to show a distinct pattern characterized by heightened conflict monitoring around ~ 200–350 ms post-target, inhibiting subsequent emotional processing and yielding no reliable behavioral differences. Accordingly, we expected effects corresponding to the N2/P2 complex with a fronto-central distribution rather than a posterior EPN. On this basis, we predicted that positive emojis would elicit larger late positivity (P300/LPP complex) than neutral emojis, indexing facilitated attention and response preparation, whereas negative versus neutral primes would chiefly modulate earlier, control-related N2/P2 activity without later effects. Although we did not expect a valence × face-status interaction in behavioral results, such an interaction might appear in the ERPs, with valence effects more pronounced for face than non-face primes. Finally, because prime–target semantic congruency was not manipulated, we anticipated no reliable N400 differences.

### Method

This study was approved by the Research Ethics Committee (CETO) of the Faculty of Arts, University of Groningen (ID: 99201577) on March 19, 2024 in accordance with the Declaration of Helsinki. All participants provided written informed consent prior to participation, including consent for the publication of study findings and the experimental data in anonymized or aggregated form.

#### Participants

Thirty participants were initially recruited for this experiment; one was excluded due to left-handedness, three due to error rates above chance level in the lexical decision task, and two due to artifacts in EEG data. The final sample included 24 participants (15 women and 9 men), aged 19 to 47 years (*M* = 28.9, *SD* = 7.3). All were either native speakers (*n* = 5) or proficient in English (age of acquisition ranged between 5 and 18 years; *M* = 10.5, *SD* = 6.4), right-handed according to the Edinburgh Handedness Inventory^86^ and reported no history of neurological or psychiatric disorders. All had normal or corrected-to-normal vision. Each participant provided written informed consent and received €20 for their participation. The study was approved by the Research Ethics Review Committee (CETO) at the University of Groningen.

#### Materials

All materials and associated analysis scripts are available at https://osf.io/rxzdn/?view_only=fbb1d33e339149b3bda6b4d34baccfaa.

To ensure a consistent experience across different platforms and emoji vendors, we selected a total of 192 emojis from OpenMoji 13.0 (2024), an independent emoji library assumed to be unfamiliar to all participants to minimize vendor-specific appearance confounds. These emojis were divided into six conditions (32 per condition): positive face, neutral face, negative face, positive non-face, neutral non-face, and negative non-face. Selection was based on valence ratings from the Emoji-SP norms database^87^, face and non-face emoji sets did not differ in frequency (*F*(1, 188) = 4.11, *p =*.180), familiarity (*F*(1, 188) = 0.23, *p =*.679), visual complexity (*F*(1, 188) = 2.39, *p =*.262), clarity (*F*(1, 188) = 11.28, *p =*.078), valence (*F*(1, 188) = 0.15, *p =*.739), or arousal (*F*(1, 188) = 0.35, *p =*.613). Across the three valence groups, there were no significant differences in frequency (*F*(2, 188) = 0.70, *p =*.587), familiarity (*F*(2, 188) = 1.12, *p =*. 472), visual complexity (*F*(2, 188) = 1.87, *p =*.348), and clarity (*F*(2, 188) = 2.95, *p =*.253), whereas valence (*F*(2, 188) = 381.31, *p =*.003) and arousal (*F*(2, 188) = 310.82, *p =*.003), differed as intended. Furthermore, all emojis were processed using imager package^88^ to adjust luminance and RMS contrast levels and to blur their edges. The contrast of the emojis remained constant over time, matched between the conditions. Supplementary Table S2 summarizes the psycholinguistic and visual properties of the emoji stimuli.

For the lexical decision task, 64 neutral words were selected based on valence, arousal, and dominance scores obtained from the NRC Lexicon^89^ included in the textdata R package^90^. Additionally, 64 nonwords from the ARC Nonword Database^91^ were included as filler targets. These nonwords were matched to the real words in length (*t*(99.24) = 1.69, *p* =.095), orthographic neighborhood size (*t*(125.84) = 0.42, *p* =.672), bigram frequency (*t*(122.38) = 1.31, *p* =.193), and orthographic Levenshtein distance (*t*(125.90) = 0.53, *p* =.599), calculated using the vwr R package^92^. Supplementary Table S3 summarizes these psycholinguistic properties.

Each word or nonword target was paired with an emoji prime in every prime condition. EmoTag^93^ was used to control for word–emoji co-occurrence frequencies, aiming to minimize potential semantic or associative effects between the prime and target (see Supplementary Table S1 for the descriptives). There were no differences between face and non-face (*F*(1, 188) = 2.49, *p* =.116), as well as between valence conditions (*F*(2, 188) = 0.99, *p* =.373). The final set of prime–target pairs was divided into two lists of 384 items each, ensuring that each emoji appeared twice per list (paired once with a word and once with a nonword), and that each word and nonword appeared three times, each time in a different condition. All estimated properties of emojis, words, and their pairings were matched across conditions and lists.

#### Procedure

Participants were first acquainted with the laboratory environment and screened regarding their handedness; they then signed an informed consent form, and the EEG cap with electrodes was mounted. The experiment was programmed in E-Prime 3.0 and presented on an Iiyama monitor (1920 × 1080, 60 Hz, 32-bit color). The mean brightness of the monitor was set to approximately 60 cd/m^2^ (*L*_min_ = 0.001 cd/m^2^, *L*_max_ = 120.41 cd/m^2^), with a viewing distance of 60 cm. All stimuli were presented on a black background; words were displayed in a white, monospaced Consolas font (25 pt), with each character subtending approximately 0.75° of visual angle. Emojis were scaled to 72 × 72 px, corresponding to about 1.9° of visual angle per emoji.

Prior to the main experiment, participants completed an emoji familiarization task, during which each prime emoji was presented once in randomized order. On each trial, participants provided a valence judgment (positive or negative) for the single presented emoji. This familiarization task served to standardize exposure to the OpenMoji rendering and reduce potential “pop-out” effects in the main task. It also allowed us to calibrate the EEG setup. Our initial valence assignments were based on the Emoji-SP norms^87^ (collected using Facebook-style emojis), and differences in emoji appearance across platforms can lead to different interpretations^94–96^. To address this, we calculated each emoji’s valence rating from the familiarization task to confirm that it matched the intended valence category.

Before starting the lexical decision task, participants were instructed that the study concerned word processing and that their main objective was to decide whether each presented letter string was an English word or a pseudoword. They were told to disregard the emojis presented briefly before each target. Responses were to be made as quickly and accurately as possible via button presses. Response button assignments (1 or 3 on the numeric keypad) to the index or middle finger of the right hand were counterbalanced across participants. The main task was preceded by a reminder displayed on the monitor and three practice trials. The order of trial presentation was randomized.

**Fig. 1:**
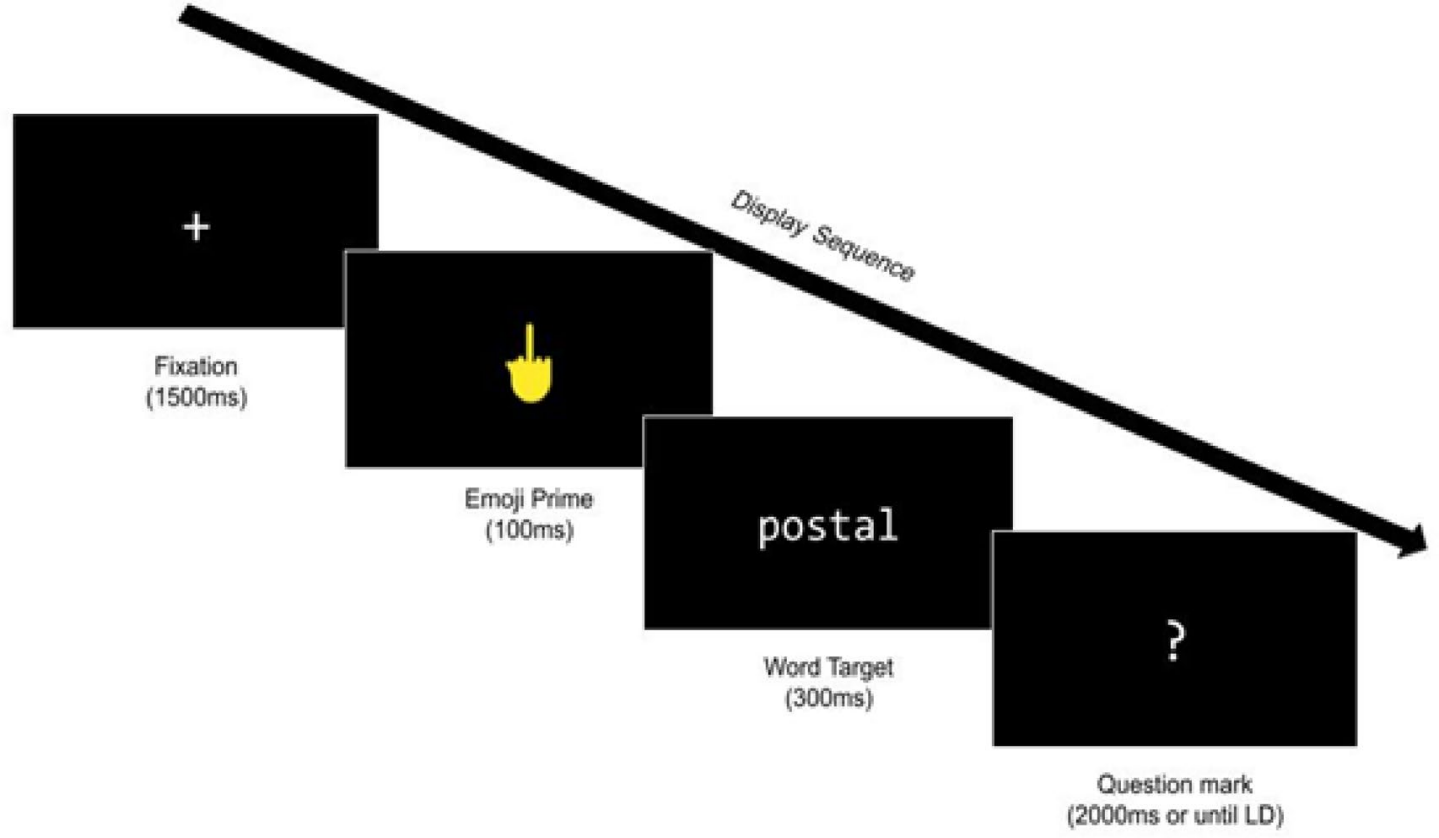
The trial sequence of emoji-primed lexical decision in Experiment 1.

Each trial started with a fixation cross for 1500 ms, followed by a 100-ms emoji prime. Immediately after prime offset, the target (word or pseudoword) was presented for 300 ms. A question mark then appeared for 2000 ms or until the participant responded. The entire recording session lasted approximately 20 min. An illustration of the trial sequence is provided in Fig. 1.

#### EEG recording

EEG data were recorded from 32 scalp channels using a WaveGuard Original cap, an EEGO-Lab system, and an eeg mylab amplifier^97^. CPz served as the online reference electrode, and the sampling rate was 500 Hz. Electrode impedances were kept below 10 kΩ. Vertical electrooculograms (VEOG) were recorded above the left eye for monitoring eye blinks.

EEG data were processed using Brain Vision Analyzer 2.3^98^. The signal was filtered between 0.1 Hz (high-pass) and 30 Hz (low-pass) with a 24 dB/oct slope, notch filtered at 50 Hz following fft inspection and re-referenced to the average of both mastoids. Eye-movement artifacts were corrected using an Independent Component Analysis (ICA) approach. Additional artifacts were mitigated by interpolating noisy channels (in total, 5 channels in 5 participants).

For stimulus-locked averages, filtered EEG data were segmented from 100 ms before target onset (word or pseudoword) to 800 ms post-target onset. The interval of 100 ms before the prime onset (i.e., from − 200 to − 100 ms before the target onset) served as the baseline. For response-locked averages, filtered data were considered from 800 ms before the participant’s button press (word–nonword decision) to 200 ms thereafter. Baseline correction was applied using the 1200–1100 ms prior to the response, provided the prime onset occurred after the correction.

For each participant, only correctly answered trials were averaged by condition, and artifacts were automatically flagged if amplitudes surpassed ± 100 µV or activity fell below 0.5 µV. Following these rejection steps, an average of 22.3% of trials were excluded from stimuli-locked data (23.6% of positive face, 20.4% of positive non-face, 21.9% of neutral face, 19.4% of neutral non-face, 24.0% of negative face and 24.5% of negative non-face trials), with no difference in the rejection rate (*F*(5, 138) = 0.64, *p =*.672). From response-locked data, an average of 18.5% was excluded (19.8% of positive face, 18.9% of positive non-face, 19.4% of neutral face, 13.8% of neutral non-face, 19.0% of negative face and 19.8% of negative non-face trials) with no difference in the rejection rate (*F*(5, 138) = 0.37, *p =*.871). Two participants were excluded due to excessive noise or artifacts, as more than 40% of their trials were lost.

#### Analyses

The data analysis plan was pre-registered (https://aspredicted.org/zs3k-kr7w.pdf). EEG data were averaged across conditions, segmented into 50-ms time bins and along with behavioral data exported to R^99^ for the statistical analyses.

##### Behavioral analyses

Reaction times and accuracy from the lexical decision task were combined into Inverse Efficiency Scores (IES) and analyzed using a linear mixed-effects regression model, fitted with the lme4 package^100^. Plots were generated using sjPlot^101^. Because continuous measures violated normality assumptions, Box–Cox transformations^102^ were applied via the MASS package^103^. Trials with residuals exceeding ± 2.5 standard deviations^104^ were trimmed, removing approximately 1.7% of the data. Fixed effects included emoji valence, face status, and their interaction. Planned contrasts were implemented using the hypr package^105^. Maximal random-effects structures^106^ with random intercepts and slopes for participants and items were initially tested and then sequentially simplified until the model converged^107^.

##### EEG analyses

Scalp electrodes were grouped into nine regions of interest (ROIs) based on visual inspection. To assess the topographical distributions and hemispheric differences of relevant effects in a hypothesis-independent way, we selected bilaterally symmetrical clusters of electrodes from condition-independent grand-average data^108^. We averaged the signals within each ROI to increase the signal-to-noise ratio and to reduce the number of statistical comparisons, thereby minimizing family-wise error^109^.

The midline central ROI included six electrodes (FC1, Cz, FC2, CP1, CP2, CPz), while the two electrodes were included into midline anterior (Fz, Fpz) and midline posterior (Pz, POz) ROIs. The remaining six lateral ROIs each contained three electrodes: left anterior (F7, F3, Fp1), right anterior (F4, F8, Fp2), left central (FC5, C3, CP5), right central (C4, FC6, CP6), left posterior (P7, P3, O1), and right posterior (P4, P8, O2).

To capture both the topographical distributions and hemispheric differences of the components, two global repeated-measures ANOVAs were run for each time window: one for lateral ROIs and one for midline ROIs using the afex package^110^. Lateral ANOVAs included within-subject factors of emoji valence (positive vs. neutral vs. negative), face status (face vs. non-face), hemisphere (left vs. right), and anteriority (anterior vs. central vs. posterior). Midline ANOVAs included all these factors except the hemisphere. Greenhouse–Geisser^111^ correction was applied when necessary, and effect sizes are reported as partial eta square (ηp²). For significant or marginally significant (*p* <.1) effects and interactions involving valence or face status, follow-up planned comparisons were performed using multiplicity-adjusted treatment-versus-control contrasts in the emmeans package^112^.

### Results

#### Behavioral results

In line with the pre-registered analysis plan, we calculated Inverse Efficiency Scores (IES) to account for speed–accuracy trade-offs^113^. IES were derived by dividing mean reaction time by the proportion of correct responses and serve as a standard efficiency measure combining accuracy and latency. Reaction times, error rates and IES for the lexical decisions are shown in Supplementary Fig. S4.

The linear mixed-effects model for IES revealed a significant facilitative effect of positive valence, indicating that trials preceded by positive emojis yielded lower IES values compared to neutral primes (*b* = −0.001, *SE* = 0.001, *t*(4177) = −2.33, *p* =.020). Additionally, negative valence approached significance (*b* = 0.001, *SE* = 0.001, *t*(4177) = 1.90, *p* =.057), suggesting a tendency toward reduced efficiency after negative compared to neutral primes. In contrast, neither face-status (*b* = −0.001 *SE* = 0.001, *t*(2295) = −0.35, *p* =.728), nor interactions between positive valence and face-status (*b* = −0.001, *SE* = 0.001, *t*(4177) = −0.62, *p* =.537) or negative valence and face-status (*b* = 0.001, *SE* = 0.001, *t*(4177) = 0.63, *p* =.529) reached significance. In addition to the pre-registered IES analysis, we conducted exploratory analyses of error rates and reaction times separately, maintaining the original model structure (see Supplementary Table S5).

#### ERP results

Supplementary Table S6 summarizes main effects and interactions of Experiment 1, and Supplementary Table S7 lists results of the follow-up pairwise comparisons.

##### Stimulus-locked analysis

**Fig. 2:**
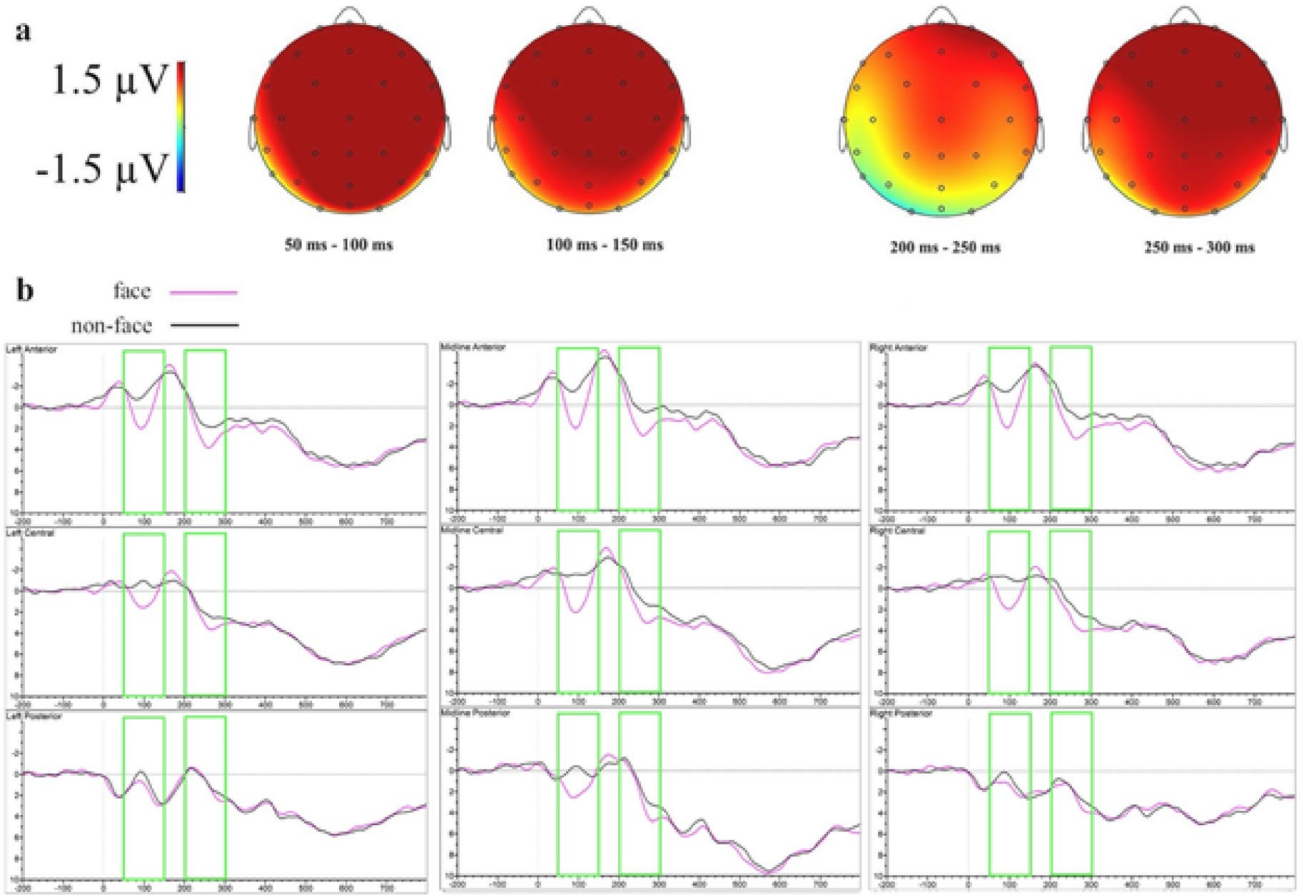
Difference topographies and ERP waveforms of the effects of prime face-status in the stimulus-locked analysis at clustered sensors in Experiment 1. (**a**) Topographic map of the significant effects of face-status (ERPs to face primes minus ERPs to non-face primes). (**b**) Grand-averaged ERP waveforms to words preceded by face and non-face emojis.

**Fig. 3:**
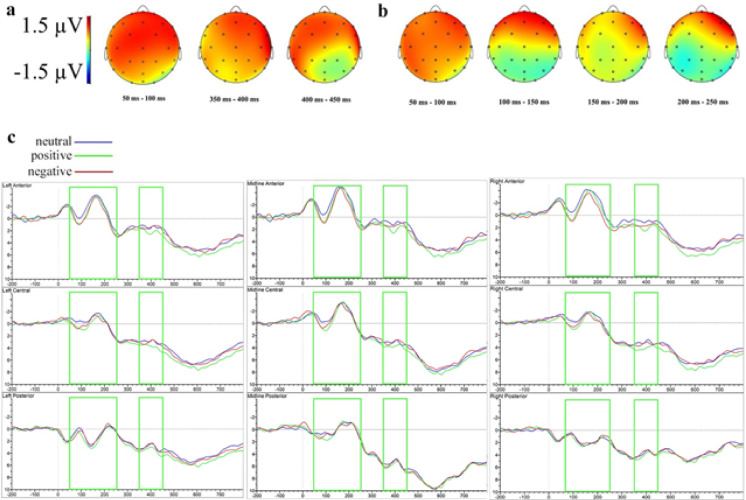
Difference topographies and ERP waveforms of the effects of prime valence in the stimulus-locked analysis at clustered sensors in Experiment 1. (**a**) Topographic map of the significant effects of positive valence (ERPs to positive primes minus ERPs to neutral primes). (**b**) Topographic map of the significant effects of negative valence (ERPs to negative primes minus ERPs to neutral primes). (**c**) Grand-averaged ERP waveforms to words preceded by neutral, positive and negative emojis.

Figure 2 illustrates the topographic maps and ERP waveforms depicting the prime face-status effects. Prime valence effects in the stimulus-locked analysis are shown in Fig. 3.

In the earliest significant time window (50–100 ms), main effects of face-status emerged at both lateral (*F*(1, 23) = 12.95, *p* =.002, ηₚ² = 0.36) and midline (*F*(1, 23) = 24.58, *p* <.001, ηₚ² = 0.52) sites, reflecting greater positivity for face compared to non-face primes (*t*(23) = 3.60, *p* =.002 at lateral; *t*(23) = 4.96, *p* <.001 at midline). A main effect of valence emerged at lateral electrodes (*F*(1.95, 44.87) = 3.81, *p* =.031, ηₚ² = 0.14), showing greater positivity for negative compared to neutral primes (*t*(23) = 2.77, *p* =.021). A significant interaction between face-status and anteriority at lateral sites (*F*(1.24, 28.51) = 12.19, *p* <.001, ηₚ² = 0.35) indicated greater positivity for face primes at anterior (*t*(23) = 4.90, *p* <.001) and central (*t*(23) = 3.76, *p* =.001) electrodes. An interaction involving valence, anteriority, and hemisphere (*F*(2.71, 62.33) = 3.43, *p* =.026, ηₚ² = 0.13) revealed positive primes elicited greater positivity than neutral primes at right anterior sites (*t*(23) = 2.43, *p* =.044), while negative primes showed increased positivity over central left (*t*(23) = 2.68, *p* =.025), central right (*t*(23) = 2.64, *p* =.028) and anterior right (*t*(23) = 2.51, *p* =.038) electrodes. A marginally significant interaction involving valence, face-status, and anteriority (*F*(2.22, 51.16) = 2.71, *p* =.071, ηₚ² = 0.11) indicated greater positivity for face over non-face items in central (*t*(23) = 2.11, *p* =.046) and anterior (*t*(23) = 3.27, *p* =.003) regions for neutral primes, central (*t*(23) = 3.20, *p* =.004) and anterior (*t*(23) = 2.92, *p* =.008) regions for positive primes, and central (*t*(23) = 2.85, *p* =.009) and anterior (*t*(23) = 2.92, *p* =.008) regions for negative primes.

The subsequent time window (100–150 ms) revealed significant main effects of face-status at both lateral (*F*(1, 23) = 18.86, *p* <.001, ηₚ² = 0.45) and midline sites (*F*(1, 23) = 26.24, *p* <.001, ηₚ² = 0.53), with face primes eliciting greater positivity compared to non-face primes (*t*(23) = 4.34, *p* <.001 at lateral; *t*(23) = 5.12, *p* <.001 at midline). Significant interactions between face-status and anteriority were observed at lateral (*F*(1.22, 28.03) = 11.30, *p* =.001, ηₚ² = 0.33) and midline (*F*(1.58, 36.35) = 4.93, *p* =.019, ηₚ² = 0.18) sites, indicating greater positivity for face primes at lateral anterior (*t*(23) = 4.99, *p* <.001), lateral central (*t*(23) = 4.24, *p* <.001), midline anterior (*t*(23) = 5.28, *p* <.001), midline central (*t*(23) = 5.11, *p* <.001), and midline posterior (*t*(23) = 3.16, *p* =.004) electrodes. Significant interactions between valence and anteriority were found at both lateral (*F*(2.18, 50.22) = 5.67, *p* =.005, ηₚ² = 0.20) and midline (*F*(2.26, 52.03) = 3.95, *p* =.021, ηₚ² = 0.15) electrodes. Post hoc tests indicated that negative primes showed greater positivity compared to neutral primes at anterior lateral (*t*(23) = 2.87, *p* =.017) and anterior midline (*t*(23) = 2.52, *p* =.036) electrodes.

In the next time window (150–200 ms), marginally significant interaction involving valence and anteriority (*F*(2.28, 52.41) = 2.71, *p* =.069, ηₚ² = 0.11) was observed at lateral sites. Post hoc analyses revealed significantly greater positivity for negative compared to neutral primes at anterior electrodes (*t*(23) = 2.66, *p* =.027).

Between 200 and 250 ms, significant valence by anteriority interactions emerged at both lateral (*F*(2.21, 50.91) = 5.70, *p* =.005, ηₚ² = 0.20) and midline sites (*F*(2.21, 50.79) = 3.60, *p* =.031, ηₚ² = 0.14), reflecting greater positivity for negative compared to neutral primes at lateral anterior (*t*(23) = 3.33, *p* =.006) and midline anterior (*t*(23) = 2.72, *p* =.024) electrodes. Additionally, marginal interaction involving valence, anteriority, and hemisphere (*F*(2.80, 64.45) = 2.27, *p* =.093, ηₚ² = 0.09) emerged at lateral electrodes. Post hoc analyses demonstrated significantly greater positivity for negative compared to neutral primes at anterior right (*t*(23) = 3.76, *p* =.002) and anterior left (*t*(23) = 2.66, *p* =.027) electrodes.

In the 250–300 ms window, a significant main effect of face-status was observed at midline sites (*F*(1, 23) = 7.38, *p* =.012, ηₚ² = 0.24), indicating greater positivity for face compared to non-face primes (*t*(23) = 2.72, *p* =.012 midline). Face-status also significantly interacted with anteriority at lateral electrodes (*F*(1.28, 29.53) = 4.64, *p* =.031, ηₚ² = 0.17), with greater positivity for face primes at anterior regions (*t*(23) = 2.56, *p* =.018).

The subsequent time window (350–400 ms) showed a marginally significant interaction between valence and anteriority at midline electrodes (*F*(2.40, 55.28) = 2.86, *p* =.056, ηₚ² = 0.11), with post hoc analyses revealing significantly greater positivity for positive compared to neutral primes at anterior sites (*t*(23) = 2.56, *p* =.033).

Between 400 and 450 ms, a significant valence by anteriority interaction at midline sites (*F*(2.65, 60.95) = 4.39, *p* =.010, ηₚ² = 0.16) indicated greater positivity for positive versus neutral primes at anterior midline sites (*t*(23) = 2.43, *p* =.044).

Finally, significant valence-by-anteriority interactions emerged at midline electrodes in the 450–500 ms (*F*(2.44, 56.11) = 3.32, *p* =.034, ηₚ² = 0.13) and 550–600 ms (*F*(1.89, 43.56) = 3.88, *p* =.030, ηₚ² = 0.14) windows, though no significant post hoc comparisons were found.

##### Response-locked analysis

**Fig. 4:**
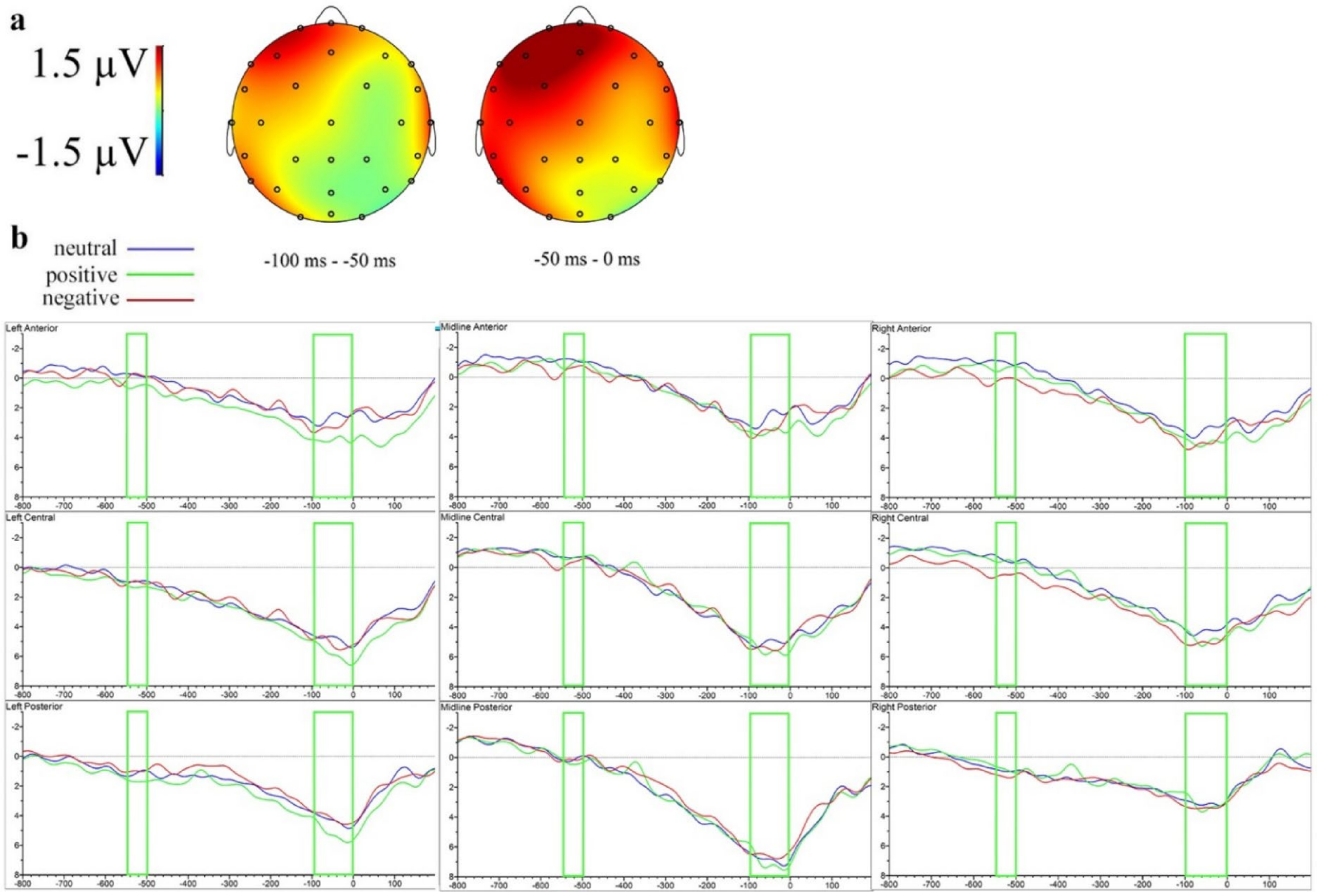
Difference topographies and ERP waveforms of the effects of prime valence in the response-locked analysis at clustered sensors in Experiment 1. (**a**) Topographic map of the significant effects of positive valence (ERPs to positive primes minus ERPs to neutral primes). (**b**) Grand-averaged ERP waveforms to responses preceded by neutral, positive and negative emojis.

Figure 4 illustrates the valence effects in the response-locked analysis. Supplementary Fig. S8 illustrates the response-locked waveforms following face and non-face primes.

An interaction between valence and hemisphere at lateral electrodes was significant in the earliest examined time window, − 800 to − 750 ms (*F*(1.87, 43.11) = 5.85, *p* =.007, ηₚ² = 0.20), and remained significant at − 750 to − 700 ms (*F*(1.87, 43.05) = 5.46, *p* =.009, ηₚ² = 0.19); −700 to − 650 ms (*F*(1.94, 44.64) = 3.47, *p* =.041, ηₚ² = 0.13); −650 to − 600 ms (*F*(1.77, 40.82) = 6.66, *p* =.004, ηₚ² = 0.23); −600 to − 550 ms (*F*(1.77, 40.65) = 6.99, *p* =.003, ηₚ² = 0.23); and − 550 to − 500 ms (*F*(1.65, 37.91) = 8.98, *p* =.001, ηₚ² = 0.28). However, follow-up comparisons did not reveal significant differences.

Between − 550 and − 500 ms, a significant interaction between valence and face-status at midline electrodes (*F*(1.69, 38.93) = 3.99, *p* =.032, ηₚ² = 0.15) revealed greater positivity for face compared to non-face responses for both positive (*t*(23) = 2.27, *p* =.033) and negative items (*t*(23) = 2.28, *p* =.032).

The valence by hemisphere interaction at lateral sites remained significant from − 500 to − 450 ms (*F*(1.68, 38.67) = 8.94, *p* =.001, ηₚ² = 0.28); −450 to − 400 ms (*F*(1.81, 41.53) = 5.76, *p* =.008, ηₚ² = 0.20); −400 to − 350 ms (*F*(1.63, 37.43) = 7.21, *p* =.004, ηₚ² = 0.24); and − 350 to − 300 ms (*F*(1.50, 34.57) = 5.41, *p* =.015, ηₚ² = 0.19). At − 350 to − 300 ms (*F*(1.49, 34.25) = 4.10, *p* =.036, ηₚ² = 0.15), and at − 300 to − 250 ms (*F*(1.53, 35.16) = 3.77, *p* =.043, ηₚ² = 0.14), there was significant interaction between face-status and anteriority, but no differences were significant in post-hoc contrasts.

The valence by hemisphere interaction at lateral sites was significant from − 250 to − 200 ms (*F*(1.75, 40.29) = 4.09, *p* =.029, ηₚ² = 0.15), − 200 to − 150 ms (*F*(1.87, 43.10) = 5.43, *p* =.009, ηₚ² = 0.19), and − 150 to − 100 ms (*F*(1.98, 45.60) = 5.58, *p* =.007, ηₚ² = 0.20). However, follow-up comparisons again did not reveal significant differences.

In the − 100 to − 50 ms window, a significant three-way interaction of valence, face-status, and anteriority appeared at lateral sites, (*F*(2.33, 53.50) = 3.10, *p* =.046, ηₚ² = 0.12), with greater positivity for positive relative to neutral faces at posterior regions (*t*(23) = 2.56, *p* =.033).

From − 50 to 0 ms, a valence by face-status interaction was significant at midline sites, (*F*(1.93, 44.32) = 4.06, *p* =.025, ηₚ² = 0.15), reflecting greater positivity for positive faces than neutral faces, (*t*(23) = 2.43, *p* =.044).

### Discussion

The results of Experiment 1 showed that both emoji valence and face-status modulate visual word recognition. Behaviorally, positive valence significantly facilitated lexical decision performance by reducing Inverse Efficiency Scores (IES), indicating quicker and more accurate responses following positive relative to neutral primes. In contrast, neither face-status nor negative valence influenced behavioral outcomes, consistent with prior evidence highlighting selective behavioral facilitation for positive stimuli in affective priming paradigms^17,18,74^.

In the stimulus-locked ERPs, face emojis elicited pronounced positivity as early as 50–150 ms post-target onset, suggesting that target processing was influenced by the face manipulation as reflected by the modulation of the P1 component. Face emojis continued to show greater positivity than non-face emojis in the 250–300 ms interval, likely reflecting overlapping P2 and P3a effects. Supporting this interpretation, interactions between face-status and anteriority in both time windows indicated a bilateral fronto-central topography. These findings align with our hypothesized 100–300 ms window and indicate a strong face-status influence on early target processing, presumably reflecting perceptual–attentional modulation. However, the lack of face-status effects in behavioral and response-locked data suggests that while facial features capture early attentional resources, they do not necessarily impact the higher-order mechanisms involved in lexical decision-making.

Valence effects partially confirmed our preregistered predictions. The stimulus-locked ERPs revealed an early positivity in the 50–100 ms window for both positive and negative primes, likely reflecting a P1 effect. In line with previous findings from emoji priming^73,74^, this suggests that the rapid perceptual mechanisms engaged in processing of emotional content at the earliest stages may be insensitive to valence. However, for positive primes, this effect was maximal over right anterior regions, whereas for negative primes it extended to bilateral central sites, indicating a broader distribution. Negative valence also showed greater anterior positivity between 100 and 250 ms, yet did not affect motor preparation or behavioral performance. This pattern, consistent with our predictions of N2/P2 modulations, indicates that negative emotional content may initiate a distinct processing route that prevents these early differences from influencing subsequent motor responses and behavior.

For positive effects, the absence of a clearly defined EPN modulation, in contrast to previous findings^17^, may be attributable to the neutral-target paradigm used. This indicates that the elicitation of the EPN component in affective priming may depend either on the availability of explicit emotional cues in targets or on the degree to which the prime is contextually significant and task-relevant. The later anterior ERP effect observed between 350 and 450 ms is consistent with our LPC/LPP prediction, suggesting enhanced evaluative processing triggered by positive primes. However, an alternative explanation is that this window also captures an N400-like modulation, comparable in the negative and neutral conditions but reduced for positive primes. Neutral targets can adopt affective features from an emotional context^114,115^, and in line with the “density hypothesis,” this could result in smaller N400s and facilitated lexico-semantic processing via positive affective priming^21^. Since our design did not manipulate prime–target semantic relatedness, and enhanced late positivity can overlap in time and scalp distribution with an N400 reduction, we cannot decisively decompose the observed effect. Future work that orthogonally manipulates semantic relatedness and affective context will be needed to arbitrate between these accounts.

In the response-locked waveforms, positive face and negative face primes elicited greater positivity compared to non-face items from − 550 ms to − 500 ms, suggesting early recognition of emotional content in face-like stimuli. Moreover, only positive face primes produced a positivity from − 100 ms to 0 ms before the response, suggesting that facilitated response preparation on positively cued trials underlies the observed performance advantage and that valence effects on response preparation are largely produced by face emojis. This interpretation aligns with prior research demonstrating that late ERP components^116,117^ are optimally captured through response-locked analyses, and LPC specifically^118^ since it is time-locked to the decision rather than to stimulus onset. In the context of our findings, the facilitation of lexical decision-making by positive primes may be driven by decision-related processes reflected in the LPC/LPP. This underscores the importance of using response-locked ERP analyses to fully capture the dynamics of affective priming effects.

While the results of Experiment 1 provide evidence for the impact of emoji valence and face-status on lexical decision-making, several limitations warrant further investigation. Positive emoji primes elicited robust behavioral facilitation accompanied by ERP modulations reflecting attentional engagement (P1), emotional evaluation (LPC/LPP), and response preparation. However, it remains unclear if any of these ERP effects can be attributed specifically to lexico-semantic processing in lexical decision. Moreover, due to the supraliminal presentation of primes, participants had full conscious awareness of the emoji cues, leaving the degree of automaticity in these effects unclear. Negative emoji primes produced ERPs suggestive of attentional engagement (P1) and conflict monitoring (P2), raising the possibility that the absence of behavioral effects resulted from top-down conscious control processes. Prior findings demonstrated that fearful faces under CFS break suppression faster than neutral faces^119^, suggesting that in processing of subliminal negative information top-down control might be reduced. Additionally, while early ERP modulations (P1) observed for face-status primes parallel those typically reported for human faces^120,121^, face-like emojis are not strictly human faces and it remains unclear whether they utilize the same route for subliminal processing reported in human face studies under CFS. While face emojis and facial expressions share certain recognition pathways^65^ and elicit similar N170 responses relative to non-face stimuli^66^, emoji recognition does not necessarily involve the fusiform face area (specific to human faces) and may rely more on the occipital face area and object-related regions^67^. Moreover, recent work distinguishes neural responses to face versus non-face emojis^72^, suggesting that face emojis (rather than non-face ones) could recruit later-stage evaluative processes rather than strictly lexical or semantic routes. To address these concerns, Experiment 2 was designed to examine which ERP markers of valence and face processing emerge under limited conscious perception via CFS masking of emoji primes.

## Experiment 2

Experiment 2 aimed to investigate whether face-status and valence effects observed in Experiment 1 persist under subliminal conditions. We utilized the same emoji-primed lexical decision task as in Experiment 1, employing Continuous Flash Suppression (CFS) masking to ensure primes remained invisible, explicitly targeting subliminal processing. Following the pre-registered design (https://aspredicted.org/zs3k-kr7w.pdf), we expected subliminal primes would modulate ERP components associated with early visual and attentional processing, although these effects were anticipated to differ qualitatively from those observed with supraliminal primes. Specifically, we conducted three planned comparisons mirroring Experiment 1: face versus non-face emojis, positive versus neutral, and negative versus neutral emojis, to systematically evaluate their subliminal influence on lexical decision-making and associated neural responses.

### Method

#### Participants

Participants were the same individuals who took part in Experiment 1 but were randomly assigned a different stimulus list here (List 2 if they had received List 1 in Experiment 1, and vice versa). The order of the two experiments was counterbalanced across participants. Data from one participant were removed due to EEG artifacts, resulting in a final sample of 23 participants.

#### Materials

All materials and associated analysis scripts are available at https://osf.io/rxzdn/?view_only=fbb1d33e339149b3bda6b4d34baccfaa.

The same sets of emojis used in Experiment 1 were employed here, and prime–target pairs, as well as the overall number of trials, remained identical. Chromatic Mondrian pattern masks served as the CFS stimuli. These masks were generated with the CFS-crafter toolbox^122^, with random RGB values (0–255) and varied square sizes and positions. Relative to the emojis (see Supplementary Table S2), the masks had greater luminance (*M* = 0.38, *SD* = 0.08; *t*(305.18) = 6.26, *p* <.001), higher RMS contrast (*M* = 0.31, *SD* = 0.04; *t*(381.99) = 5.51, *p* <.001), and increased edge density (*M* = 0.27, *SD* = 0.04; *t*(381.38) = 27.21, *p* <.001). The size of the Mondrian components ranged from 0.02° to 0.60°, while the font and emojis retained the same size as in Experiment 1 (0.78° × 0.78° for words and 1.70° × 1.70° for emojis).

#### Procedure

For binocular fusion^123,124^, the monitor continuously displayed two symmetrical white frames (6° × 10° of visual angle) on a black background, spaced 1° apart, and a white central fixation cross (0.4˚ × 0.4˚) in the center of each frame. Participants, with head position fixed at approximately 60 cm from the monitor, viewed the stimuli through a Sokkisha MS16 mirror stereoscope, enabling presentation of each frame individually to the corresponding eye.

Since CFS efficacy depends substantially on eye dominance, with stronger masking observed when the suppressed stimulus is presented to the participant’s non-dominant eye^125,126^, the task setup was configured depending on participants’ eye dominance. Following Johansson et al.^127^, participants’ eye dominance was determined using two established tests: the Miles^128^ and Porta^129^, as cited in^130^, tests. These assessments indicated that four participants were left-eye dominant, and the remaining 19 participants were right-eye dominant.

Prior to the main task, participants completed a pre-test to confirm that they could not consciously see the stimuli. This forced-choice discrimination task resembled the familiarization task in Experiment 1 (i.e., evaluating whether emojis were positive or negative), but the emojis were now masked with CFS. Participants were asked to press the spacebar if they perceived any image other than the flashing grids; if not, they guessed whether the unseen emoji was positive or negative. This step validated participants’ eye dominance, allowed adjustments to the setup (e.g., monitor brightness, stereoscope alignment, viewing distance), and ensured that the stimuli were sufficiently suppressed to function as subliminal primes. In this discrimination task, 33.0% of trials were reported as visible, and after removing those trials, accuracy remained at chance (*M* = 52.7%, *SD* = 8.4%), confirming successful masking.

**Fig. 5:**
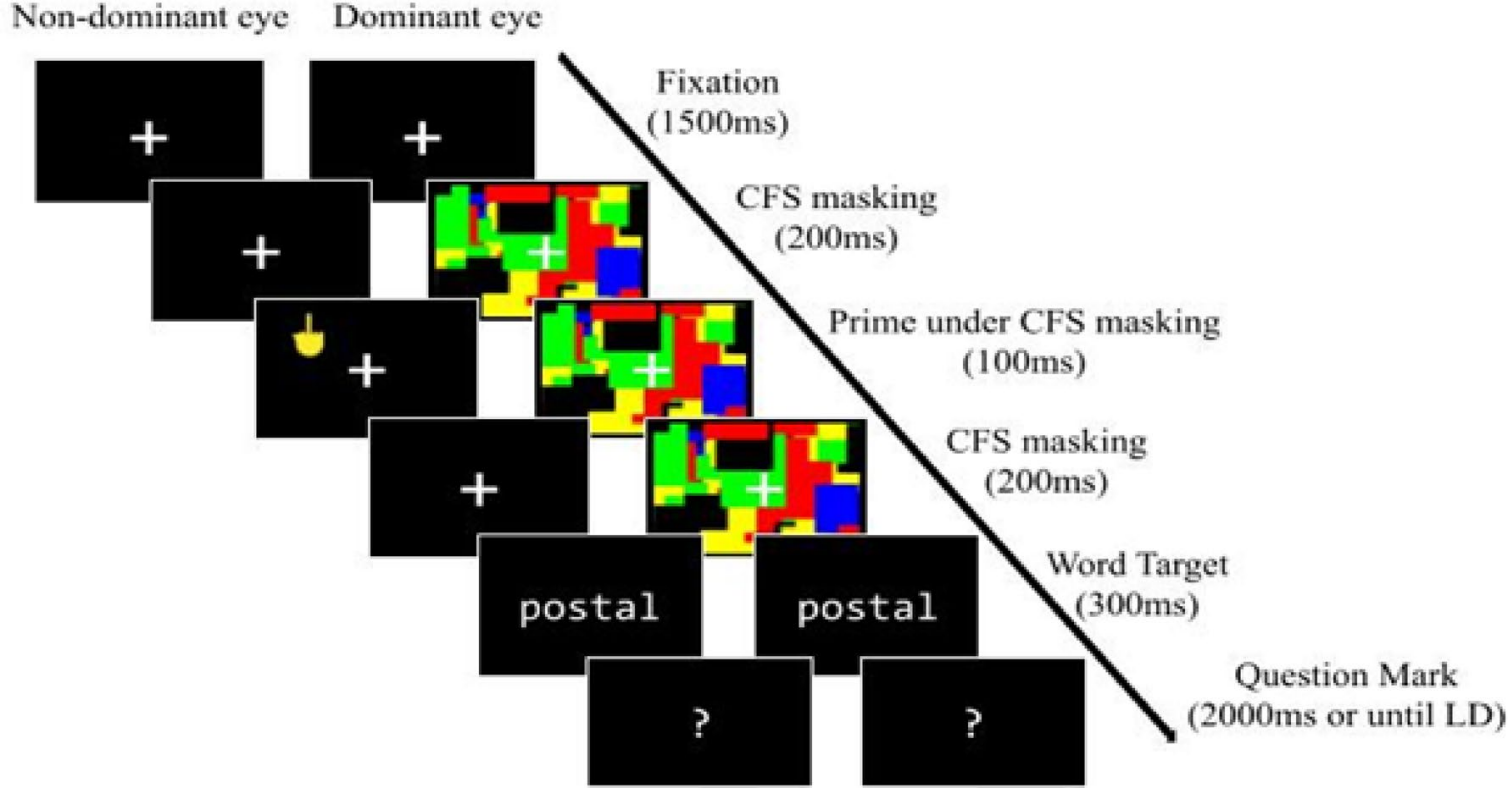
The trial sequence of CFS-masked emoji-primed lexical decision in Experiment 2.

Instructions for the main task largely matched those from Experiment 1, except participants were told to press the spacebar whenever they perceived any image beyond the Mondrian patterns, in which case the trial would be skipped and excluded from further analysis. As depicted in Fig. 5, each trial began with a fixation cross, identically presented to each eye for 1500 ms. Subsequently, high-contrast Mondrian masks occupied the entire frame presented to the dominant eye for 500 ms, refreshing at 10 Hz. Simultaneously, the emoji prime was briefly presented for 100 ms in one of the four corners of the non-dominant eye’s frame, starting 200 ms after mask onset and ending 200 ms before mask offset. Following Kim et al.^64^, this timing ensured minimal prime visibility. The prime location was counterbalanced across conditions and pseudo-randomly varied between trials, minimizing potential attentional biases toward any particular spatial location^131–133^, thus reducing the risk that sustained spatial attention could facilitate prime visibility or alter subliminal processing^134–136^. Afterward, the target word or pseudoword was then presented to both eyes for 300 ms, followed by a question mark for up to 2000 ms or until the participant responded. After three practice trials, the main task was presented in a single randomized block. The entire session lasted approximately 40 min.

#### EEG recording

EEG was recorded following the same specifications as in Experiment 1, with baseline correction adjusted for the new trial timing. For stimulus-locked averages, baseline correction applied to the 100 ms before prime onset (i.e., from − 400 to − 300 ms before target onset). For response-locked averages, the interval of 900–800 ms before the response served as the baseline. Figure 5.

#### Analysis

Trials in which participants reported perceiving the prime were excluded (20.2% of the data), per the CFS design. Other analysis steps, including artifact rejection thresholds, reference procedures, and region-of-interest definitions, were identical to those in Experiment 1. Following exclusion of incorrectly responded trials and artifact rejection, 27.4% of trials were excluded from stimulus-locked data (28.1% of positive face, 27.3% of positive non-face, 28.3% of neutral face, 24.5% of neutral non-face, 30.3% of negative face and 25.7% of negative non-face trials), with no difference in the rejection rate (*F*(5, 132) = 0.37, *p =*.867). From response-locked data, an average of 19.5% was excluded (20.5% of positive face, 17.3% of positive non-face, 20.5% of neutral face, 17.1% of neutral non-face, 23.4% of negative face and 18.3% of negative non-face trials) with no difference in the rejection rate (*F*(5, 132) = 0.54, *p =*.749). One participant was excluded due to excessive noise or artifacts, as more than 40% of their trials were lost. Behavioral data were again analyzed using Inverse Efficiency Scores, trimming ± 2.5 *SD* residuals (~ 2.6% of trials).

### Results

#### Behavioral results

Reaction times, error rates and Inverse Efficiency Scores (IES) for the lexical decisions under CFS are shown in Supplementary Fig. S9. The linear mixed-effects model revealed a significant inhibition effect of negative valence, with higher IES scores for targets preceded by negative compared to neutral emojis (*b* = 0.005, *SE* = 0.002, *t*(3626) = 2.83, *p* =.005). Positive valence approached significance, suggesting a trend towards improved efficiency after positive compared to neutral primes (*b* = −0.003, *SE* = 0.002, *t*(3622) = −1.92, *p* =.056). Neither face-status (*b* = 0.001, *SE* = 0.003, *t*(561.8) = 0.24, *p* =.811) nor interactions between positive valence and face-status (*b* = 0.002, *SE* = 0.003, *t*(3622) = 0.78, *p* =.433) or negative valence and face-status (*b* = 0.001, *SE* = 0.003, *t*(3625) = 0.46, *p* =.644) reached significance.

As in Experiment 1, additional exploratory analyses were conducted separately on error rates and reaction times (see Supplementary Table S10).

#### ERP results

Supplementary Table S11 summarizes main effects and interactions of Experiment 2, and Supplementary Table S12 lists results of the follow-up pairwise comparisons.

##### Stimulus-locked analysis

**Fig. 6:**
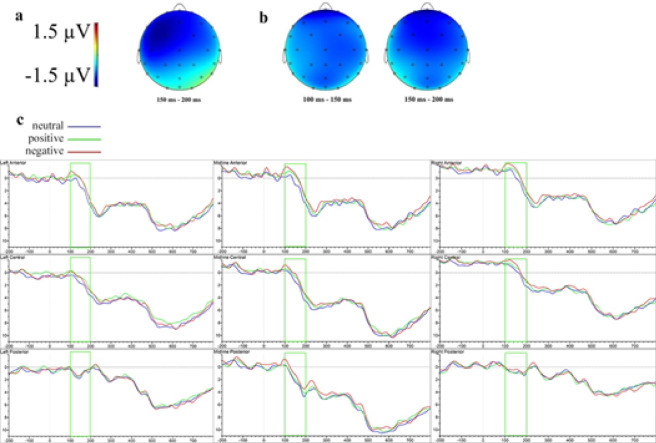
Difference topographies and ERP waveforms of the effects of prime valence under CFS in the stimulus-locked analysis at clustered sensors in Experiment 2. (**a**) Topographic map of the significant effects of positive valence under CFS (ERPs to positive primes minus ERPs to neutral primes). (**b**) Topographic map of the significant effects of negative valence under CFS (ERPs to negative primes minus ERPs to neutral primes). (**c**) Grand-averaged ERP waveforms to words preceded by neutral, positive and negative emojis under CFS.

Figure 6 shows the ERP modulations associated with positive and negative valence under CFS. The waveforms following face and non-face primes in the stimulus-locked data are presented in Supplementary Fig. S13.

In the earliest significant time window (100–150 ms), a marginal main effect of valence at midline electrodes (*F*(1.91, 41.98) = 2.53, *p* =.094, ηₚ² = 0.10) indicated significantly greater negativity for negative compared to neutral primes (*t*(22) = −2.40, *p* =.047).

Between 150 and 200 ms, a marginally significant valence effect emerged at midline electrodes (*F*(1.80, 39.57) = 3.32, *p* =.051, ηₚ² = 0.13), with negative primes eliciting greater negativity compared to neutral primes (*t*(22) = −2.48, *p* =.040). Additionally, a significant four-way interaction involving valence, face-status, anteriority, and hemisphere was observed at lateral electrodes (*F*(2.87, 63.04) = 3.12, *p* =.034, ηₚ² = 0.12), showing greater negativity for positive compared to neutral primes specifically for face stimuli at central left electrodes (*t*(22) = −2.67, *p* =.027). Similarly, significant four-way interactions emerged at lateral regions between 200 and 250 ms (*F*(2.62, 57.70) = 4.34, *p* =.011, ηₚ² = 0.17) and 300–350 ms (*F*(2.42, 53.33) = 3.15, *p* =.042, ηₚ² = 0.13), but post hoc comparisons were not significant.

In the 350–400 ms window, a marginal four-way interaction (*F*(2.73, 60.00) = 2.81, *p* =.052, ηₚ² = 0.11) emerged, with post hoc analyses showing significantly greater negativity for positive versus neutral primes for non-face items at anterior left electrodes (*t*(22) = 2.56, *p* =.034).

Between 400 and 450 ms, a significant four-way interaction (*F*(3.04, 66.81) = 6.50, *p* <.001, ηₚ² = 0.23) revealed greater negativity for negative compared to neutral primes at anterior left electrodes specifically for face stimuli (*t*(22) = −2.44, *p* =.043). The 450–500 ms window also displayed a significant four-way interaction at lateral sites (*F*(3.13, 68.79) = 5.51, *p* =.002, ηₚ² = 0.20), but no significant post hoc differences emerged.

From 550 to 600 ms, a significant valence-by-face-status interaction emerged at midline electrodes (*F*(1.91, 41.97) = 3.69, *p* =.035, ηₚ² = 0.14), with face stimuli eliciting greater positivity compared to non-face stimuli for neutral primes (*t*(22) = 2.25, *p* =.035). A marginal four-way interaction was also observed at lateral sites (*F*(2.37, 52.22) = 2.49, *p* =.083, ηₚ² = 0.10), with post hoc analyses showing increased negativity for negative compared to neutral primes for face items at anterior left electrodes (*t*(22) = −2.57, *p* =.033).

From 600 to 650 ms, a significant interaction between valence and face-status emerged at midline electrodes (*F*(1.97, 43.33) = 3.57, *p* =.037, ηₚ² = 0.14), indicating greater positivity for face over non-face neutral primes (*t*(22) = 2.52, *p* =.020). A marginal four-way interaction was also observed at lateral sites (*F*(2.19, 48.23) = 2.54, *p* =.085, ηₚ² = 0.10), with increased negativity for negative compared to neutral primes specifically for face stimuli at anterior left sites (*t*(22) = −2.72, *p* =.024).

From 700 to 750 ms, a marginal four-way interaction at lateral sites (*F*(2.88, 63.39) = 2.72, *p* =.054, ηₚ² = 0.11) revealed greater negativity for negative compared to neutral face stimuli at anterior left electrodes (*t*(22) = −2.50, *p* =.039). Similarly, between 750 and 800 ms, a significant four-way interaction at lateral sites (*F*(2.53, 55.61) = 3.54, *p* =.026, ηₚ² = 0.14) again revealed greater negativity for negative compared to neutral face items at anterior left electrodes (*t*(22) = −2.41, *p* =.047).

##### Response-locked analysis

**Fig. 7:**
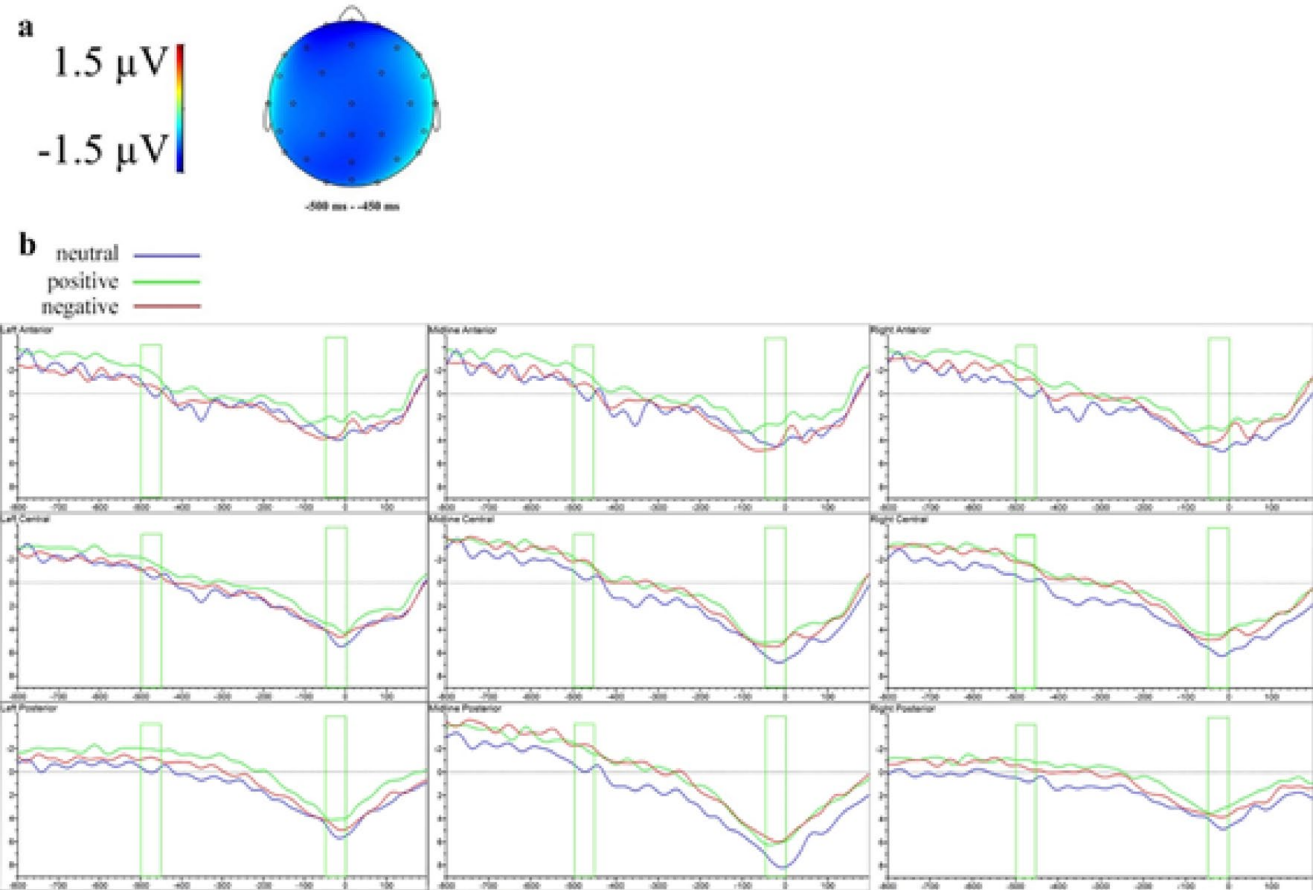
Difference topographies and ERP waveforms of the effects of prime valence under CFS in the response-locked analysis at clustered sensors in Experiment 2. (**a**) Topographic map of the significant effects of negative valence under CFS (ERPs to negative primes minus ERPs to neutral primes). (**b**) Grand-averaged ERP waveforms to responses preceded by neutral, positive and negative emojis under CFS.

Figure 7 illustrates valence effects in the response-locked ERP analysis under CFS. Supplementary Fig. S14 demonstrates the waveforms for face and non-face primes.

Between − 700 and − 650 ms, a significant interaction between face-status and hemisphere (*F*(1, 22) = 5.10, *p* =.034, ηₚ² = 0.19) emerged at lateral electrodes, but no significant post hoc differences were observed.

Between − 500 and − 450 ms, a marginal effect of valence at midline regions (*F*(1.92, 42.32) = 3.16, *p* =.055, ηₚ² = 0.13) indicated significantly greater negativity for negative compared to neutral responses (*t*(22) = −2.56, *p* =.034). Additionally, a significant valence-by-face-status interaction emerged at midline electrodes (*F*(1.80, 39.61) = 3.66, *p* =.039, ηₚ² = 0.14), demonstrating greater negativity for negative versus neutral responses specifically for face stimuli (*t*(22) = −3.06, *p* =.011).

Between − 300 and − 250 ms, a marginal valence-by-face-status interaction at midline sites (*F*(1.64, 36.14) = 3.23, *p* =.060, ηₚ² = 0.13) revealed greater positivity for positive compared to neutral responses for non-face stimuli (*t*(22) = 2.41, *p* =.047).

Similarly, between − 250 and − 200 ms, a marginal valence-by-face-status interaction at midline electrodes (*F*(1.82, 40.03) = 3.26, *p* =.053, ηₚ² = 0.13) demonstrated greater positivity for positive versus neutral responses specifically for non-face stimuli (*t*(22) = 2.58, *p* =.032).

In the − 150 to − 100 ms interval, significant interactions involving face-status and hemisphere (*F*(1, 22) = 5.54, *p* =.028, ηₚ² = 0.20) and face-status and anteriority at midline electrodes (*F*(1.37, 30.11) = 3.91, *p* =.045, ηₚ² = 0.15) indicated face-status modulations without significant post hoc differences.

In the interval immediately preceding response execution (− 50 to 0 ms), a significant valence-by-face-status interaction at midline electrodes (*F*(1.75, 38.42) = 5.19, *p* =.013, ηₚ² = 0.19) revealed greater negativity for negative compared to neutral responses specifically for face stimuli (*t*(22) = −3.26, *p* =.007).

### Discussion

Experiment 2 examined whether valence- and face-related modulations observed in Experiment 1 persist under continuous flash suppression (CFS). Our preliminary forced-choice discrimination task confirmed the effectiveness of CFS in suppressing awareness of color emojis. Awareness checks indicated that participants rarely perceived masked emojis; reported awareness decreased from 33% (when emojis were the direct target) to 20% (when serving as primes), suggesting suppression effectiveness is sensitive to task parameters. This demonstrates that rectangular high-contrast Mondrian masks provide robust suppression for circular face emojis. Although feature-selective suppression accounts suggest that masks matching certain target features (e.g., shape, color) might yield stronger suppression^137,138^ the empirical evidence is mixed^126,139^. Some studies describe binocular suppression as generally a non-selective process^140^, indicating that edge density and overall mask–target property matching may be more critical for successful CFS than geometric similarity^141^. Indeed, our findings favor this latter perspective, as rectangular masks suppressed color emojis at least as efficiently as the circular masks often used in CFS studies with human facial expressions (e.g^142–144^.

No significant face-status effects emerged in either the behavioral or electrophysiological measures under CFS. In contrast to the pronounced face-related modulations of Experiment 1, subliminal prime presentation disrupted perceptual–attentional engagement by face-like cues. These results imply that conscious awareness is essential for face-like emoji features to modulate lexical decision processes.

Valence patterns also diverged from Experiment 1. No behavioral facilitation for positive primes emerged, implying that the positivity advantage observed under supraliminal conditions requires conscious awareness. Consistent with this interpretation, positive primes did not demonstrate a modulation in a later LPP/LPC time-window nor the response-locked effect observed in Experiment 1, further underscoring the role of conscious processing in driving the positivity advantage. In contrast, a response-locked modulations were demonstrated for negative primes, accompanied by an inhibitory effect on behavioral performance, indicating a valence-specific influence on motor response preparation. This aligns with previous findings of subliminal negative-valence inhibition effects^18,22,29,85^.

Moreover, a four-way interaction emerged in the stimulus-locked ERPs at 400–450 ms and 700–800 ms, characterized by greater left-anterior negativity for negative-face versus neutral-face primes. Although face-status showed no main effect under CFS, this pattern suggests that when negative content is conveyed by a face emoji, it can exert a selective influence on neural responses. One possibility is that, under suppression, cues from negative faces retain privileged access to evaluative or control-related processes^119,145,146^. Another, not mutually exclusive, explanation is that partial feature leakage or variability in suppression depth differentially affects face versus non-face primes^141,143^. Consistent with this interpretation, negative-face emojis in our data were most likely to break suppression and produced more artifacts and errors than other conditions. Moreover, the sustained left-hemisphere, anterior locus of the effect suggests biased lexico–semantic rather than purely emotional processing^147,148^. Because this interaction did not generalize to behavior and was not preregistered, we interpret it cautiously and highlight it as a promising target for follow-up work that manipulates facial configuration and arousal while tightly controlling suppression depth.

Furthermore, compared to overt priming in Experiment 1, the ERP effects in similar time windows reversed direction under CFS. In Experiment 1, both positive and negative compared to neutral primes elicited a positive-going effect in the P1 window (50–150 ms after target onset), whereas in Experiment 2, positive and negative primes compared to neutral ones demonstrated a negative-going difference within 100–200 ms post-target onset. A similar reversal under CFS was reported by Yang et al.^149^, who found a reversed N400 effect when comparing conscious to suppressed priming. Hence, our results suggest that reversed priming effects may be a broader phenomenon under CFS. Although the specific mechanisms remain unclear, this pattern underscores the possibility that conscious and nonconscious priming engage different neural routes or timing, warranting further investigation.

Overall, Experiment 2 demonstrates that face-like features and positive emotional content lose their modulatory strength under CFS masking, whereas negative valence retains a degree of early cortical impact, as evidenced by robust stimulus- and response-locked negativities and inhibition of lexical decisions.

## General discussion

Across two experiments, we investigated how emoji primes varying in emotional valence (positive, neutral, negative) and face-status (face vs. non-face) influence lexical decision-making and underlying neural correlates. Our primary aims were threefold: (a) to determine whether positive or negative emojis confer facilitation or interference under supraliminal conditions, (b) to assess the role of face-like features in modulating early attentional mechanisms and later decision processes, and (c) to examine whether these effects persist when prime awareness is minimized using continuous flash suppression (CFS). Overall, we observed a clear positivity advantage under conscious prime visibility in Experiment 1, whereas negative primes showed limited behavioral impact. In Experiment 2, under CFS masking, negative primes produced early neural modulations and an inhibitory effect on lexical decisions, while the positivity advantage vanished. Furthermore, main effect of face-status modulated early neural responses exclusively under conscious conditions, with no measurable behavioral or neural impact under CFS.

Experiment 1 revealed that consciously perceived positive primes significantly facilitated lexical decisions, whereas negative primes had minimal behavioral effect. ERP analyses indicated that both positive and negative primes elicited early attentional modulation (P1), but only positive primes engaged response-locked components associated with enhanced motor preparation, late evaluative processes (LPP), and potentially an N400 reduction. Negative primes uniquely produced a P2 modulation, indicating a distinct neural pathway potentially related to heightened conflict monitoring^13,14^. These electrophysiological patterns support a dual-pathway framework^85,150^, in which positive primes recruit later evaluative or semantic processes linked to response facilitation, while negative primes primarily affect earlier perceptual–attentional stages without necessarily influencing decision-making.

Face-status strongly influenced early and mid-latency ERP components (P1, P2, P3a) but yielded no behavioral benefit, implying that face-like emojis capture early attention without translating into faster lexical decisions. One explanation is that while facial features effectively attract early attentional resources, they do not necessarily influence the higher-order processes required for lexical decisions^151,152^. Alternatively, face-like properties might exert opposing influences on top-down and bottom-up attention^153^: facial cues may automatically enhance bottom-up processing while simultaneously distracting top-down attentional resources away from the target. As a result, these opposing forces could counteract each other, leading to null behavioral effects.

The lack of a main effect of face-status under CFS in Experiment 2 suggests that face-like features require conscious awareness to engage attentional and behavioral mechanisms. This contrasts with findings involving human faces, where certain facial features can remain accessible even under masking^39,40^. Our results align with evidence indicating distinct neural representations for emoji faces compared to human faces^66^. Specifically, face emojis may not engage the fusiform face area to the same extent as real human faces, relying instead primarily on occipital face and object-related regions^67^. Thus, activation of these areas likely depends on sufficient conscious processing.

Valence effects under CFS differed markedly from Experiment 1. ERP analyses showed significant negativities for both positive and negative primes between 150 and 200 ms, demonstrating that emotional content from emojis can be accessed under CFS. However, negative primes elicited earlier onset and more sustained negativities, accompanied by response-locked negativity and behavioral inhibition. Thus, positive primes no longer facilitated lexical decisions, whereas negative primes retained early neural impact and slowed behavioral responses. These findings support existing evidence showing an advantage for negative stimuli under subliminal conditions^154^, consistent with automatic vigilance accounts^155^. Moreover, we observed a significant interaction between valence and face-status in Experiment 2, suggesting privileged subliminal processing of negative emotional content specifically from face emojis. Additionally, ERP effects under CFS showed reversed polarity compared to conscious priming^149^, suggesting distinct neural processes engaged by subliminal versus conscious affective priming, warranting further investigation.

In sum, our findings suggest a potential dissociation between effects of positive and negative priming across varying awareness conditions. Positive emojis facilitated lexical decisions primarily when consciously perceived, engaging evaluative mechanisms dependent upon awareness. Negative emojis exerted limited behavioral effects when consciously viewed but maintained inhibitory influence under subliminal masking, indicative of more automatic (potentially subcortical) processing. Moreover, under conscious conditions, the negative stimuli interference might be suppressed by early inhibitory mechanisms, likely inactive or weakened during subliminal processing. Future research should explore this assumed dissociation directly.

Several methodological constraints should be noted. First, CFS inevitably excluded a number of trials due to partial awareness reports, which may have reduced the power to detect subtle effects in Experiment 2. Second, our familiarization task may not have fully eliminated background familiarity differences, and using the same participant pool for both experiments leaves open the possibility of familiarity or carryover effects, although our counterbalanced design aimed to mitigate this. Future studies might adopt separate samples or novel stimuli to minimize familiarity. They could also explicitly manipulate top-down versus bottom-up attention in prime detection or examine individual differences that might modulate these effects. Finally, although our task did not require explicit valence judgments, we cannot entirely exclude the possibility of a response compatibility confound, since a lexical decision ultimately involves a “yes” or “no” response that could align or conflict with the prime’s valence. For instance, positive valence might be implicitly associated with a “yes” response and negative valence with a “no” response, even in tasks not overtly focusing on emotional evaluation. Additional experimental designs (e.g., go/no-go tasks or tasks with neutral response mappings) might be warranted to fully disentangle response compatibility from purely perceptual–attentional or semantic priming.

## Conclusion

Taken together, our findings suggest distinct effects of positive and negative emoji primes on lexical decision making under varying prime-awareness conditions. Under supraliminal conditions, positive emojis yielded a clear behavioral benefit and were associated with both early (P1) and late (LPP) enhancements, as well as a response-locked positivity indicating facilitated motor response preparation. Negative emojis, by contrast, showed no overt behavioral facilitation despite eliciting early (P1, P2) electrophysiological modulations, suggesting heightened perceptual–attentional engagement that did not translate into faster decisions. When primes were masked with continuous flash suppression (CFS), face-like cues from emojis no longer influenced behavior or early ERPs, indicating that they require conscious awareness for their salience to emerge. Positive emojis lost their advantage altogether under these conditions, highlighting a crucial dependence on awareness for positivity-driven facilitation. Negative primes, however, remained partially potent under masking, displaying robust early neural activity and an inhibition of behavioral responses. Overall, these results emphasize that conscious perception is a key moderator of positivity bias, while negative valence exerts a more automatic influence that remains effective under limited conscious processing.

## Supplementary Information

Below is the link to the electronic supplementary material.

Supplementary Material 1

## Data Availability

The materials, analysis scripts, and datasets generated and analyzed during the current study are available in the OSF repository [https://osf.io/rxzdn/?view_only=fbb1d33e339149b3bda6b4d34baccfaa]. The preregistration is available at [https://aspredicted.org/zs3k-kr7w.pdf].
