# Supplementary material for: The impact of awareness on affective emoji priming in visual word recognition
Source: Sci Rep. 2026 Jan 5;16:631. doi: 10.1038/s41598-025-34117-w (PMC12905443; doi:10.1038/s41598-025-34117-w)

Supplementary Information

Supplementary Table S1

Examples of Prime-Target Pairs across Experiments, and Means and Standard Deviations for the Co-Occurrence Frequencies

Condition	Example	Co-occurrence frequencies		
		N	*M*	*SD*
Face positive	😊 postal	64	0.64	1.28
Non-face positive	🎉 postal	64	0.55	1.46
Face neutral	👱 postal	64	0.70	1.66
Non-face neutral	👞 postal	64	0.25	0.76
Face negative	😟 postal	64	0.25	0.78
Non-face negative	💔 postal	64	0.77	1.58

Note. Emoji stimuli were extracted from OpenMoji 14.0 – the open-source emoji and icon project. License: CC BY-SA 4.0.

Supplementary Table S2

Means and Standard Deviations for Psycholinguistic and Visual Properties of Emoji Stimuli

Metric	Face						Non-face					
	Negative		Neutral		Positive		Negative		Neutral		Positive	
	M	*SD*	*M*	*SD*	*M*	*SD*	*M*	*SD*	*M*	*SD*	*M*	*SD*
Frequency [a]	2.45	1.19	1.88	1.33	1.93	1.31	1.49	0.84	1.36	0.69	1.86	0.98
Familiarity [a]	3.11	1.35	2.66	1.49	2.77	1.56	2.63	1.13	2.35	1.20	3.17	1.31
Valence [a]	3.33	0.74	5.11	0.25	6.62	0.71	3.54	0.60	5.12	0.29	6.51	0.73
Arousal [a]	5.95	0.91	4.70	0.76	4.62	0.86	5.99	0.84	4.70	0.98	4.49	1.10
Visual Complexity [a]	3.51	1.11	3.59	0.83	3.72	1.13	3.13	1.20	3.65	1.03	3.42	1.00
Clarity [a]	5.84	0.98	5.39	1.19	5.90	0.90	6.19	0.92	6.02	1.25	6.13	1.00
Luminance [b]	0.35	0.05	0.35	0.03	0.36	0.03	0.33	0.05	0.34	0.05	0.33	0.06
Contrast (RMS)[b]	0.31	0.03	0.30	0.03	0.31	0.03	0.28	0.04	0.28	0.04	0.28	0.04
Edge Density [b]	0.17	0.03	0.17	0.03	0.15	0.03	0.15	0.05	0.16	0.04	0.16	0.04

Note. Valence and arousal scores range from 1 to 9; familiarity, frequency, clarity, and visual complexity scores range from 1 to 7.

[a] Retrieved from Emoji-SP by Ferré et al. (2023).

[b] Calculated via imager package (Barthelme, 2024).

Supplementary Table S3

Means and Standard Deviations for Psycholinguistic Properties of Word and Non-Word Stimuli

Metric	Words		Non-Words	
	M	*SD*	*M*	*SD*
Number of Letters	8.16	1.99	7.84	1.22
Orthographic Neighbors [a]	0.41	0.91	0.25	0.72
Orthographic Levenshtein Distance (OLD20) [a]	2.84	0.62	2.92	0.72
Bigram Frequency [a]	27060	11565	25337	10492
Valence [b]	0.56	0.11	-	
Arousal [b]	0.44	0.17	-	
Dominance [b]	0.56	0.15	-	

Note. Valence, arousal, and dominance range from 0 (lowest) to 1 (highest).

[a] Retrieved via the vwr R package (Keuleers & Keuleers, 2013)

[b] Retrieved from the NRC Lexicon (Mohammad, 2018) in the textdata R package (Hvitfeldt, 2024).

Supplementary Figure S4

Mean Reaction Times (a), Error Rates (b) and Inverse Efficiency Scores (c) by Condition in Experiment 1

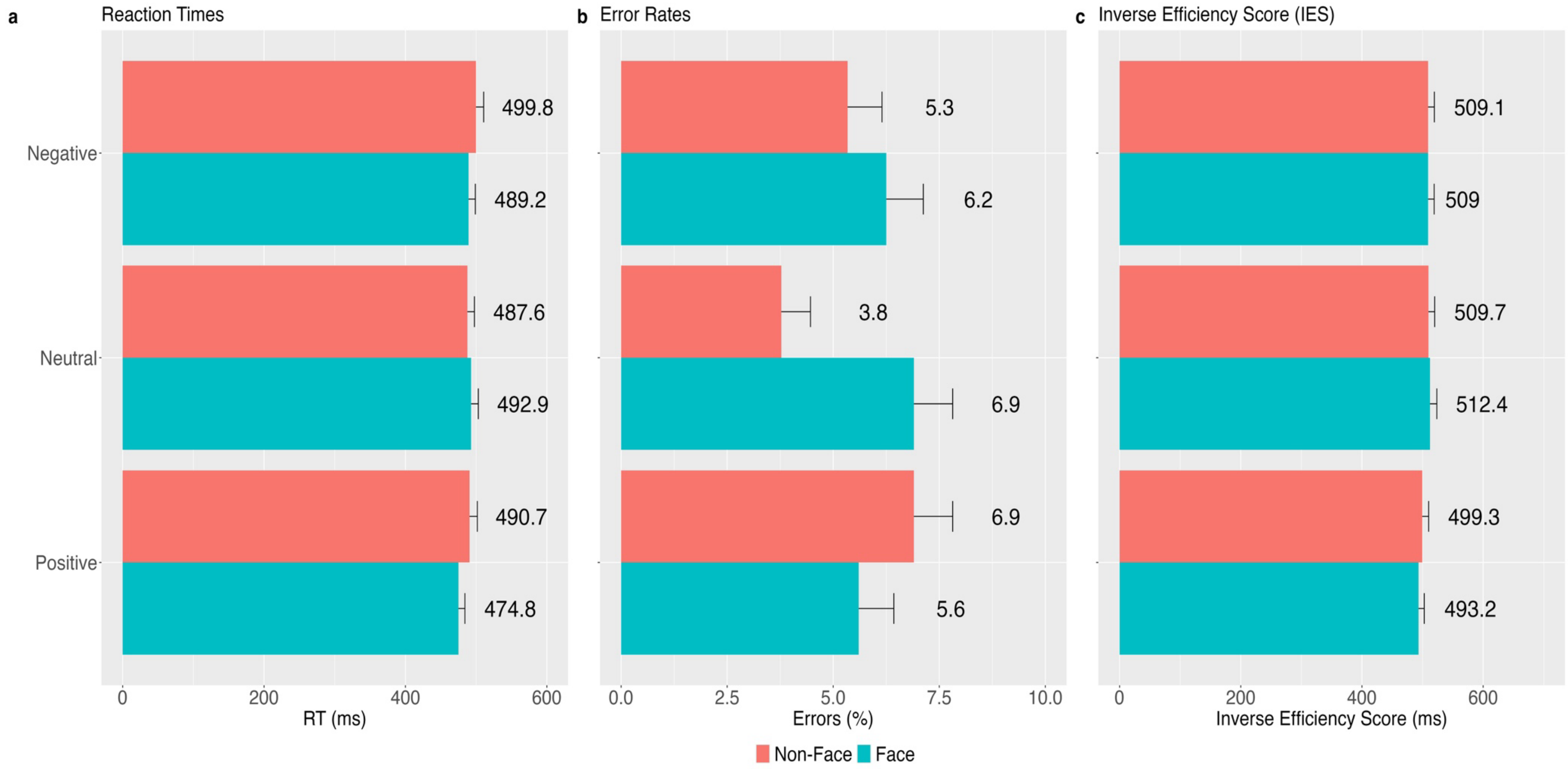

Note. Error bars represent the Standard Error of the Mean (SEM).

Supplementary Table S5

Results of Mixed Effects Models for Error Rates and Reaction Times in Experiment 1

Predictor	*β*	*SE*	*t(df) / z*	*p*
	Error rates			
Face-status	-0.32	0.18	-1.73	.085
Positive Valence	-0.11	0.09	-1.24	.216
Negative Valence	-0.02	0.09	-0.19	.851
**Face-status × Positive Valence**	**0.49**	**0.18**	**2.66**	**.008**
Face-status × Negative Valence	0.03	0.19	0.15	.882
	Reaction Times			
Face-status	-0.0002	0.0004	-0.40(2303)	.690
**Positive Valence**	**-0.0006**	**0.0002**	**-2.50(4176)**	**.012**
Negative Valence	0.0005	0.0002	1.88(4176)	.061
Face-status × Positive Valence	-0.0003	0.0005	-0.59(4176)	.556
Face-status × Negative Valence	0.0003	0.0005	0.71(4176)	.477

Note. Significant effects are indicated in boldface

Supplementary Table S6

Significant and Marginal Main Effects and Interactions in ERP analysis of Experiment 1

Time-window (ms)	Region	Effect	*F*	*dfs*	*p*	η_p^2
		Stimulus-locked				
50-100	Lateral	Valence	3.81*	1.95, 44.87	.031	.142
	Lateral	Face-status	12.95**	1, 23	.002	.36
	Lateral	Face-status: Anteriority	12.19***	1.24, 28.51	< .001	.35
	Lateral	Face-status: Hemisphere	4.01+	1, 23	.057	.15
	Lateral	Valence: Face-status: Anteriority	2.71+	2.22, 51.16	.071	.11
	Lateral	Valence: Anteriority: Hemisphere	3.43*	2.71, 62.33	.026	.13
	Midline	Face-status	24.58***	1, 23	< .001	.52
100-150	Lateral	Face-status	18.86***	1, 23	< .001	.45
	Lateral	Valence: Anteriority	5.67**	2.18, 50.22	.005	.20
	Lateral	Face-status: Anteriority	11.30**	1.22, 28.03	.001	.33
	Midline	Face-status	26.24***	1, 23	< .001	.53
	Midline	Valence: Anteriority	3.95*	2.26, 52.03	.021	.15
	Midline	Face-status: Anteriority	4.93*	1.58, 36.35	.019	.18
150-200	Lateral	Valence: Anteriority	2.71+	2.28, 52.41	.069	.11
	Lateral	Valence: Hemisphere	2.62+	1.88, 43.26	.087	.10
200-250	Lateral	Valence: Anteriority	5.70**	2.21, 50.91	.005	.20
	Lateral	Face-status: Anteriority	3.41+	1.42, 32.64	.060	.13
	Lateral	Valence: Hemisphere	2.85+	1.74, 40.12	.076	.11
	Lateral	Face-status: Hemisphere	5.29*	1, 23	.031	.19
	Lateral	Valence: Anteriority: Hemisphere	2.27+	2.80, 64.45	.093	.09
	Midline	Valence: Anteriority	3.60*	2.21, 50.79	.031	.14
	Midline	Face-status: Anteriority	3.29+	1.24, 28.55	.072	.13
250-300	Lateral	Face-status	3.85+	1, 23	.062	.14
	Lateral	Face-status: Anteriority	4.64*	1.28, 29.53	.031	.17
	Midline	Face-status	7.38*	1, 23	.012	.24
350-400	Midline	Valence: Anteriority	2.86+	2.40, 55.28	.056	.11
400-450	Lateral	Valence: Hemisphere	3.28*	1.99, 45.67	.047	.13
	Midline	Valence: Anteriority	4.39**	2.65, 60.95	.010	.16
450-500	Midline	Valence: Anteriority	3.32*	2.44, 56.11	.034	.13
550-600	Lateral	Valence: Face-status: Anteriority	2.65+	2.25, 51.71	.074	.10
	Midline	Valence: Anteriority	3.88*	1.89, 43.56	.030	.14
700-750	Lateral	Valence: Face-status	2.51+	1.98, 45.58	.093	.10
	Lateral	Valence: Hemisphere	2.87+	1.75, 40.31	.075	.11
	Midline	Valence: Face-status	2.80+	1.94, 44.57	.073	.11

Time-window (ms)	Region	Effect	*F*	*dfs*	*p*	η_p^2
	Response-locked					
-800-750	Lateral	Valence: Hemisphere	5.85**	1.87, 43.11	.007	.20
-750-700	Lateral	Valence: Hemisphere	5.46**	1.87, 43.05	.009	.19
-700-650	Lateral	Valence: Hemisphere	3.47*	1.94, 44.64	.041	.13
-650-600	Lateral	Valence: Hemisphere	6.66**	1.77, 40.82	.004	.23
-600-550	Lateral	Valence: Hemisphere	6.99**	1.77, 40.65	.003	.23
-550-500	Lateral	Valence: Hemisphere	8.98**	1.65, 37.91	.001	.28
	Midline	Valence: Face-status	3.99*	1.69, 38.93	.032	.15
-500-450	Lateral	Valence: Hemisphere	8.94**	1.68, 38.67	.001	.28
-450-400	Lateral	Valence: Hemisphere	5.76**	1.81, 41.53	.008	.20
-400-350	Lateral	Valence: Hemisphere	7.21**	1.63, 37.43	.004	.24
-350-300	Lateral	Face-status: Anteriority	4.10*	1.49, 34.25	.036	.15
	Lateral	Valence: Hemisphere	5.41*	1.50, 34.57	.015	.19
-300-250	Lateral	Face-status: Anteriority	3.77*	1.53, 35.16	.043	.14
	Lateral	Valence: Hemisphere	3.07+	1.55, 35.76	.071	.12
-250-200	Lateral	Valence: Hemisphere	4.09*	1.75, 40.29	.029	.15
-200-150	Lateral	Valence: Hemisphere	5.43**	1.87, 43.10	.009	.19
-150-100	Lateral	Valence: Hemisphere	5.58**	1.98, 45.60	.007	.20
-100-50	Lateral	Valence: Hemisphere	3.07+	1.99, 45.72	.056	.12
	Lateral	Valence: Face-status: Anteriority	3.10*	2.33, 53.50	.046	.12
-50-0	Lateral	Valence: Face-status	2.63+	1.95, 44.87	.085	.10
	Lateral	Valence: Hemisphere	2.51+	1.88, 43.29	.096	.10
	Midline	Valence: Face-status	4.06*	1.93, 44.32	.025	.15

+$p < .1$. *$p < .05$. **$p < .01$. *** $p < .001$.

Supplementary Table S7

Contrasts For Significant Planned Comparisons in ERP analysis of Experiment 1

Time-window (ms)	Region	Contrast	Estimate	Anteriority	Hemisphere	Valence/ Face-Status	*t.ratio*	*dfs*	*p*
				Stimulus-locked					
50-100	Lateral	Negative-Neutral	0.78	-	-	-	2.76	23	.021
	Lateral	Face-Nonface	1.15	-	-	-	3.60	23	.002
	Lateral	Face-Nonface	1.71	Anterior	-	-	4.90	23	< .001
	Lateral	Face-Nonface	1.39	Central	-	-	3.76	23	.001
	Lateral	Face-Nonface	0.94	-	Left	-	2.79	23	.010
	Lateral	Face-Nonface	1.36	-	Right	-	4.04	23	.001
	Lateral	Face-Nonface	1.93	Anterior	-	Neutral	3.27	23	.003
	Lateral	Face-Nonface	1.22	Central	-	Neutral	2.11	23	.046
	Lateral	Face-Nonface	1.43	Anterior	-	Positive	2.94	23	.007
	Lateral	Face-Nonface	1.56	Central	-	Positive	3.20	23	.004
	Lateral	Face-Nonface	1.78	Anterior	-	Negative	2.92	23	.008
	Lateral	Face-Nonface	1.40	Central	-	Negative	2.85	23	.009
	Lateral	Negative-Neutral	0.96	Central	Left	-	2.68	23	.025
	Lateral	Positive-Neutral	1.07	Anterior	Right	-	2.43	23	.044
	Lateral	Negative-Neutral	1.08	Anterior	Right	-	2.50	23	.037
	Lateral	Negative-Neutral	0.88	Central	Right	-	2.64	23	.028
	Midline	Face-Nonface	1.92	-	-	-	4.96	23	< .001
100-150	Lateral	Face-Nonface	1.13	-	-	-	4.34	23	< .001
	Lateral	Negative-Neutral	1.27	Anterior	-	-	2.87	23	.017

Time-window (ms)	Region	Contrast	Estimate	Anteriority	Hemisphere	Valence/ Face-Status	*t.ratio*	*dfs*	*p*
	Lateral	Face-Nonface	1.75	Anterior	-	-	4.99	23	< .001
	Lateral	Face-Nonface	1.29	Central	-	-	4.24	23	< .001
	Midline	Face-Nonface	1.58	-	-	-	5.12	23	< .001
	Midline	Negative-Neutral	1.41	Anterior	-	-	2.52	23	.036
	Midline	Face-Nonface	1.89	Anterior	-	-	5.28	23	< .001
	Midline	Face-Nonface	1.78	Central	-	-	5.11	23	< .001
	Midline	Face-Nonface	1.08	Posterior	-	-	3.16	23	.004
150-200	Lateral	Negative-Neutral	1.12	Anterior	-	-	2.66	23	.027
200-250	Lateral	Negative-Neutral	1.36	Anterior	-	-	3.33	23	.006
	Lateral	Negative-Neutral	1.06	Anterior	Left	-	2.66	23	.027
	Lateral	Negative-Neutral	1.67	Anterior	Right	-	3.76	23	.002
	Midline	Negative-Neutral	1.38	Anterior	-	-	2.72	23	.024
250-300	Lateral	Face-Nonface	1.40	Anterior	-	-	2.56	23	.018
	Midline	Face-Nonface	1.33	-	-	-	2.72	23	.012
350-400	Midline	Positive-Neutral	1.79	Anterior	-	-	2.56	23	.033
400-450	Midline	Positive-Neutral	1.84	Anterior	-	-	2.43	23	.044
	Response-locked								
-550-500	Midline	Face-Nonface	1.93	-	-	Positive	2.27	23	0.033
	Midline	Face-Nonface	1.04	-	-	Negative	2.28	23	0.032
-100-50	Lateral	Positive-Neutral	1.65	Posterior	-	Face	2.56	23	0.033
-50-0	Midline	Positive-Neutral	1.96	-	-	Face	2.43	23	0.044

Supplementary Figure S8

ERP Waveforms by Prime Face-Status in the Response-Locked Analysis at Clustered Sensors in Experiment 1

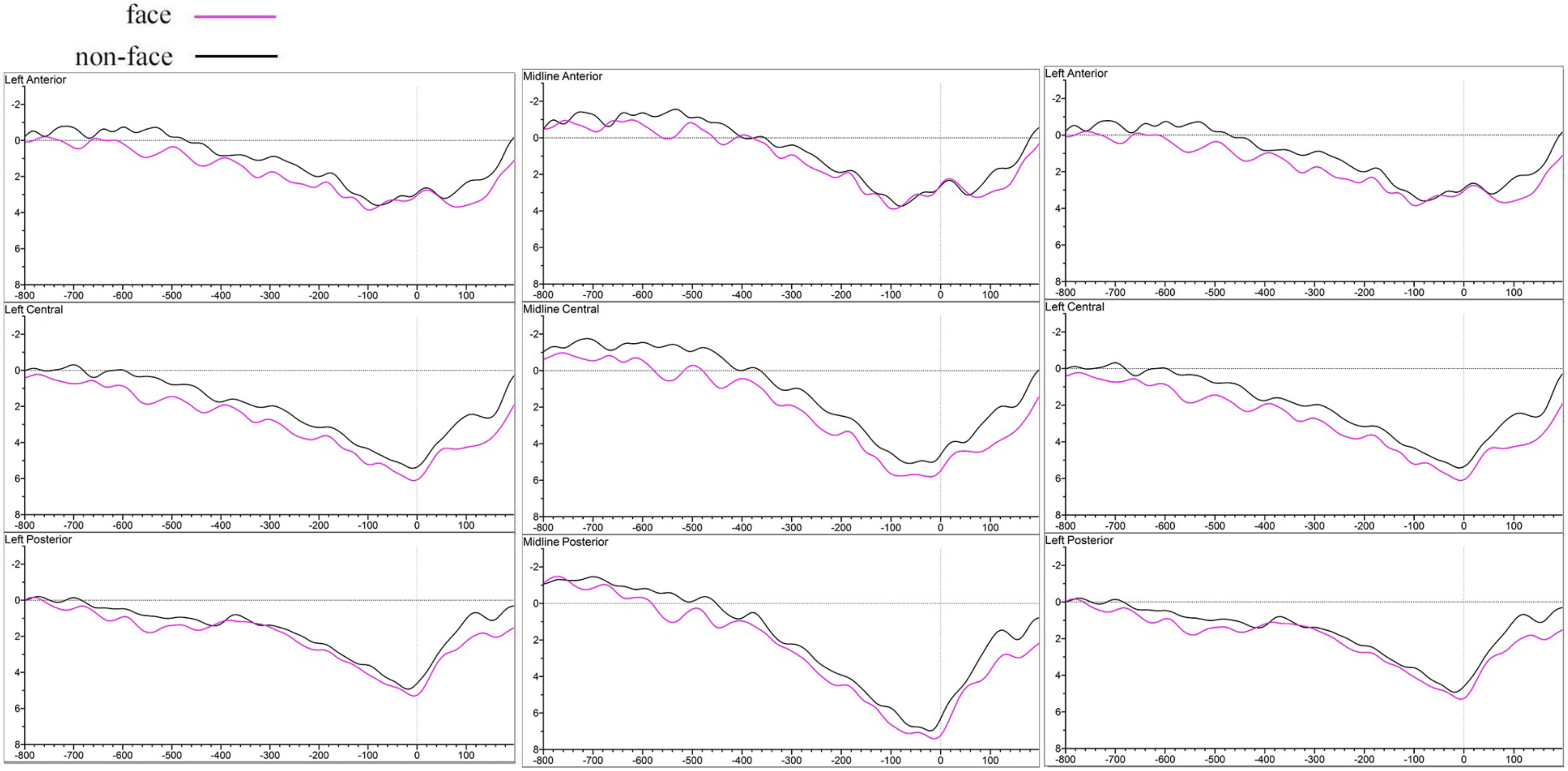

Supplementary Figure S9

Mean Reaction Times (a), Error Rates (b) and Inverse Efficiency Scores (c) by Condition in Experiment 2

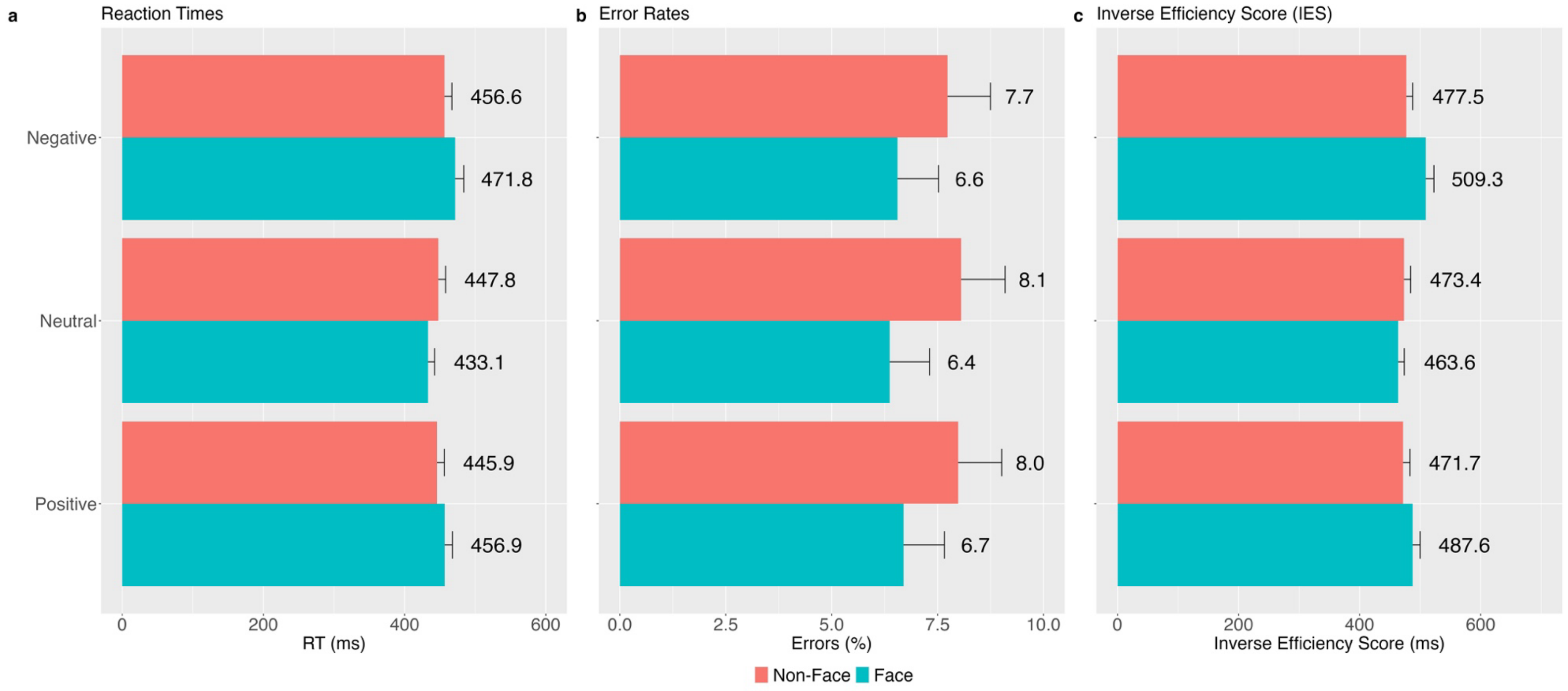

Note. Error bars represent the SEM.

Supplementary Table S10

Results of Mixed Effects Models for Error Rates and Reaction Times in Experiment 2

Predictor	*β*	*SE*	*t(df) / z*	*p*
	Error rates			
Face-status	0.089	0.160	0.56	.579
Positive Valence	-0.057	0.087	-0.66	.511
Negative Valence	0.080	0.089	0.90	.367
Face-status × Positive Valence	0.032	0.173	0.19	.852
Face-status × Negative Valence	-0.140	0.178	-0.79	.432
	Reaction Times			
Face-status	0.003	0.003	1.12(801)	.264
Positive Valence	-0.003	0.002	-1.96(3732)	.050
**Negative Valence**	**0.004**	**0.002**	**2.48(3735)**	**.013**
Face-status × Positive Valence	0.0001	0.003	0.02(3732)	.984
Face-status × Negative Valence	0.002	0.003	0.62(3735)	.538

Note. Significant effects are indicated in boldface.

Supplementary Table S11

Significant and Marginal Main Effects and Interactions in ERP analysis of Experiment 2

Time-window (ms)	Region	Effect	*F*	*dfs*	*p*	η_p^2
		Stimulus-locked				
100-150	Lateral	Valence: Hemisphere	3.14+	1.85, 40.66	.058	.13
150-200	Lateral	Valence: Face-status: Hemisphere	3.07+	1.84, 40.48	.061	.12
	Lateral	Face-status: Anteriority: Hemisphere	2.71+	1.65, 36.26	.089	.11
	Midline	Valence	2.53+	1.91, 41.98	.094	.10
	Lateral	Valence: Face-status: Anteriority: Hemisphere	3.12*	2.87, 63.04	.034	.12
	Midline	Valence	3.32+	1.80, 39.57	.051	.13
200-250	Lateral	Valence: Face-status: Anteriority: Hemisphere	4.34*	2.62, 57.70	.011	.17
	Midline	Valence	3.10+	1.72, 37.82	.064	.12
300-350	Lateral	Valence: Face-status: Anteriority: Hemisphere	3.15*	2.42, 53.33	.042	.13
350-400	Lateral	Valence: Face-status: Hemisphere	2.94+	1.66, 36.45	.075	.12
	Lateral	Valence: Face-status: Anteriority: Hemisphere	2.81+	2.73, 60.00	.052	.11
400-450	Lateral	Valence: Face-status: Anteriority: Hemisphere	6.50***	3.04, 66.81	< .001	.23
450-500	Lateral	Valence: Hemisphere	2.68+	1.93, 42.48	.082	.11
	Lateral	Valence: Face-status: Anteriority: Hemisphere	5.51**	3.13, 68.79	.002	.20
500-550	Lateral	Face-status: Anteriority: Hemisphere	2.73+	1.65, 36.19	.088	.11
	Lateral	Valence: Face-status: Anteriority: Hemisphere	2.21+	3.07, 67.43	.093	.09
550-600	Lateral	Face-status: Anteriority: Hemisphere	2.73+	1.70, 37.41	.086	.11
	Lateral	Valence: Face-status: Anteriority: Hemisphere	2.49+	2.37, 52.22	.083	.10
	Midline	Valence: Face-status	3.69*	1.91, 41.97	.035	.14
600-650	Lateral	Face-status: Anteriority: Hemisphere	2.79+	1.96, 43.17	.073	.11
	Lateral	Valence: Face-status: Anteriority: Hemisphere	2.54+	2.19, 48.23	.085	.10
	Midline	Valence: Face-status	3.57*	1.97, 43.33	.037	.14
650-700	Lateral	Valence: Hemisphere	3.21+	1.72, 37.82	.058	.13
	Lateral	Valence: Face-status: Anteriority: Hemisphere	3.15*	2.99, 65.76	.031	.13

Time-window (ms)	Region	Effect	*F*	*dfs*	*p*	η_p^2
700-750	Lateral	Valence: Face-status: Anteriority: Hemisphere	2.72+	2.88, 63.39	.054	.11
750-800	Lateral	Valence: Hemisphere	4.37*	1.63, 35.93	.026	.17
	Lateral	Valence: Face-status: Anteriority: Hemisphere	3.54*	2.53, 55.61	.026	.14
		Response-locked				
-700-650	Lateral	Face-status: Hemisphere	5.10*	1, 22	.034	.19
-550-500	Lateral	Valence: Face-status	2.52+	1.85, 40.69	.096	.10
	Midline	Valence: Face-status	2.86+	1.95, 42.92	.069	.12
-500-450	Lateral	Valence	2.45+	1.99, 43.73	.099	.10
	Lateral	Valence: Face-status	2.77+	1.81, 39.90	.080	.11
	Midline	Valence	3.16+	1.92, 42.32	.055	.13
	Midline	Valence: Face-status	3.66*	1.80, 39.61	.039	.14
-450-400	Midline	Face-status: Anteriority	2.90+	1.27, 27.93	.091	.12
-350-300	Midline	Valence: Face-status	2.73+	1.87, 41.13	.081	.11
-300-250	Lateral	Valence: Face-status	2.68+	1.66, 36.53	.091	.11
	Midline	Valence: Face-status	3.23+	1.64, 36.14	.060	.13
-250-200	Midline	Valence: Face-status	3.26+	1.82, 40.03	.053	.13
-150-100	Lateral	Face-status: Hemisphere	5.54*	1, 22	.028	.20
	Midline	Face-status: Anteriority	3.91*	1.37, 30.11	.045	.15
-100-50	Lateral	Face-status: Hemisphere	3.71+	1, 22	.067	.14
	Lateral	Face-status: Anteriority: Hemisphere	2.93+	1.41, 31.12	.084	.12
-50-0	Lateral	Valence: Face-status	4.60*	1.71, 37.67	.021	.17
	Lateral	Face-status: Hemisphere	3.61+	1, 22	.071	.14
	Lateral	Face-status: Anteriority: Hemisphere	2.86+	1.71, 37.70	.077	.12
	Midline	Valence: Face-status	5.19*	1.75, 38.42	.013	.19
	Midline	Face-status: Anteriority	2.70+	1.42, 31.18	.098	.11

+$p < .1$. *$p < .05$. **$p < .01$. *** $p < .001$.

Supplementary Table S12

Contrasts For Significant Planned Comparisons in ERP analysis of Experiment 2

Time-window (ms)	Region	Contrast	Estimate	Anteriority	Hemisphere	Valence/ Face-Status	*t.ratio*	*dfs*	*p*
				Stimulus-locked					
100-150	Midline	Negative-Neutral	-1.07	-	-		-2.40	22	.047
150-200	Lateral	Positive-Neutral	-1.52	Central	Left	Face	-2.67	22	.027
	Midline	Negative-Neutral	-1.14	-	-	-	-2.48	22	.040
350-400	Lateral	Positive-Neutral	1.57	Anterior	Left	Nonface	2.56	22	.034
400-450	Lateral	Negative-Neutral	-2.24	Anterior	Left	Face	-2.44	22	.043
	Lateral	Face-Nonface	2.16	Anterior	Left	Neutral	2.41	22	.025
550-600	Lateral	Negative-Neutral	-2.07	Anterior	Left	Face	-2.57	22	.033
	Midline	Face-Nonface	2.46	-	-	Neutral	2.25	22	.035
600-650	Lateral	Negative-Neutral	-2.11	Anterior	Left	Face	-2.72	22	.024
	Lateral	Face-Nonface	2.92	Anterior	Left	Neutral	3.18	22	.004
	Midline	Face-Nonface	2.52	-	-	Neutral	2.52	22	.020
700-750	Lateral	Negative-Neutral	-2.38	Anterior	Left	Face	-2.50	22	.039
750-800	Lateral	Negative-Neutral	-2.33	Anterior	Left	Face	-2.41	22	.047
				Response-locked					
-500-450	Midline	Negative-Neutral	-1.01	-	-	-	-2.56	22	.034
	Midline	Negative-Neutral	-2.20	-	-	Face	-3.06	22	.011
	Midline	Face-Nonface	-1.23	-	-	Negative	-2.27	22	.034
-300-250	Midline	Positive-Neutral	1.45	-	-	Nonface	2.41	22	.047
	Midline	Face-Nonface	-1.72	-	-	Positive	-2.30	22	.031

-250-200	Midline	Positive-Neutral	1.77	-	-	Nonface	2.58	22	.032
-50-0	Midline	Negative-Neutral	-2.54	-	-	Face	-3.26	22	.007
	Midline	Face-Nonface	1.77	-	-	Neutral	2.14	22	.043

Supplementary Figure S13

ERP Waveforms by Prime Face-Status in the Stimulus-Locked Analysis at Clustered Sensors in Experiment 2

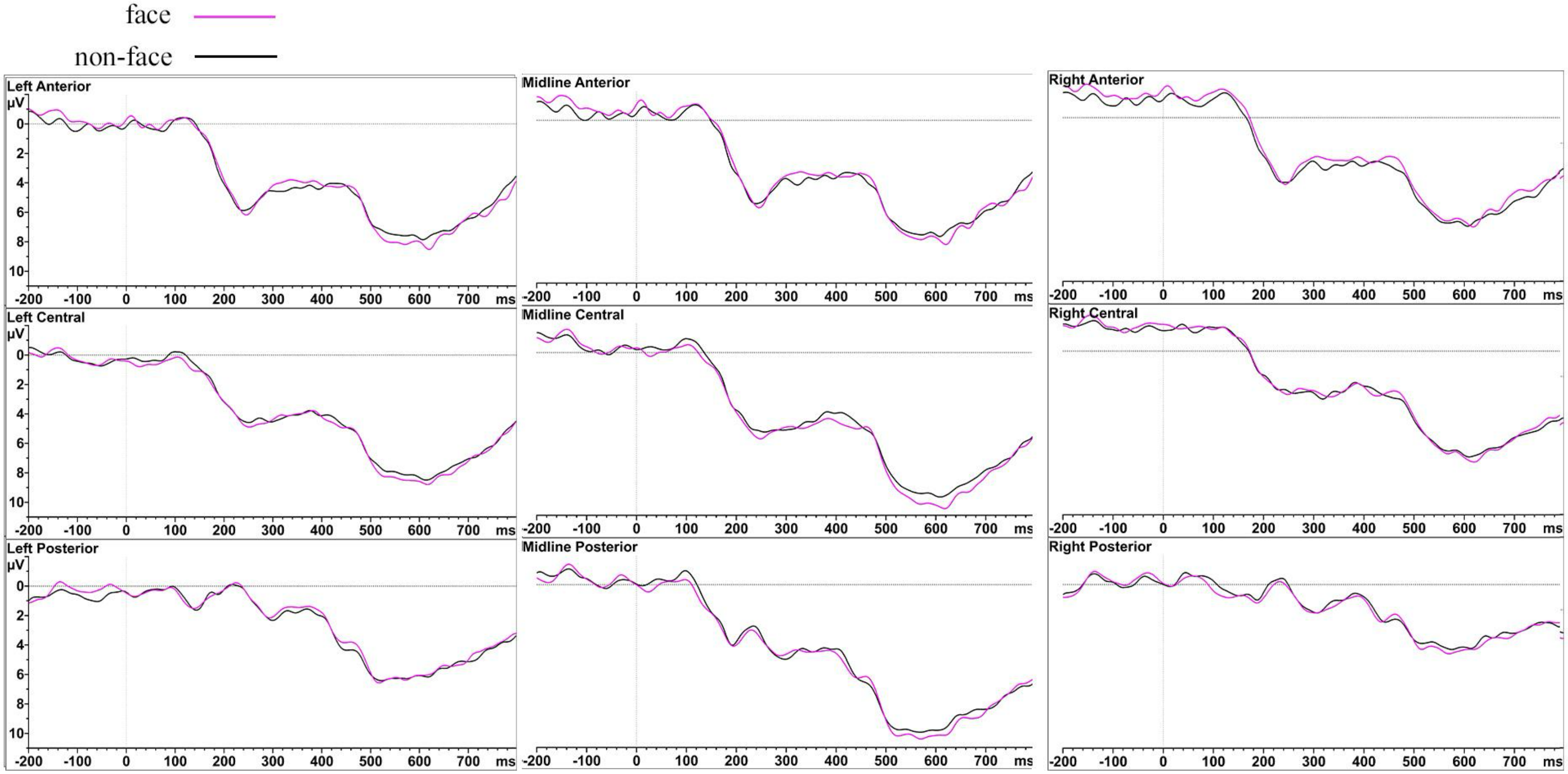

Supplementary Figure S14

ERP Waveforms by Prime Face-Status in the Response-Locked Analysis at Clustered Sensors in Experiment 2

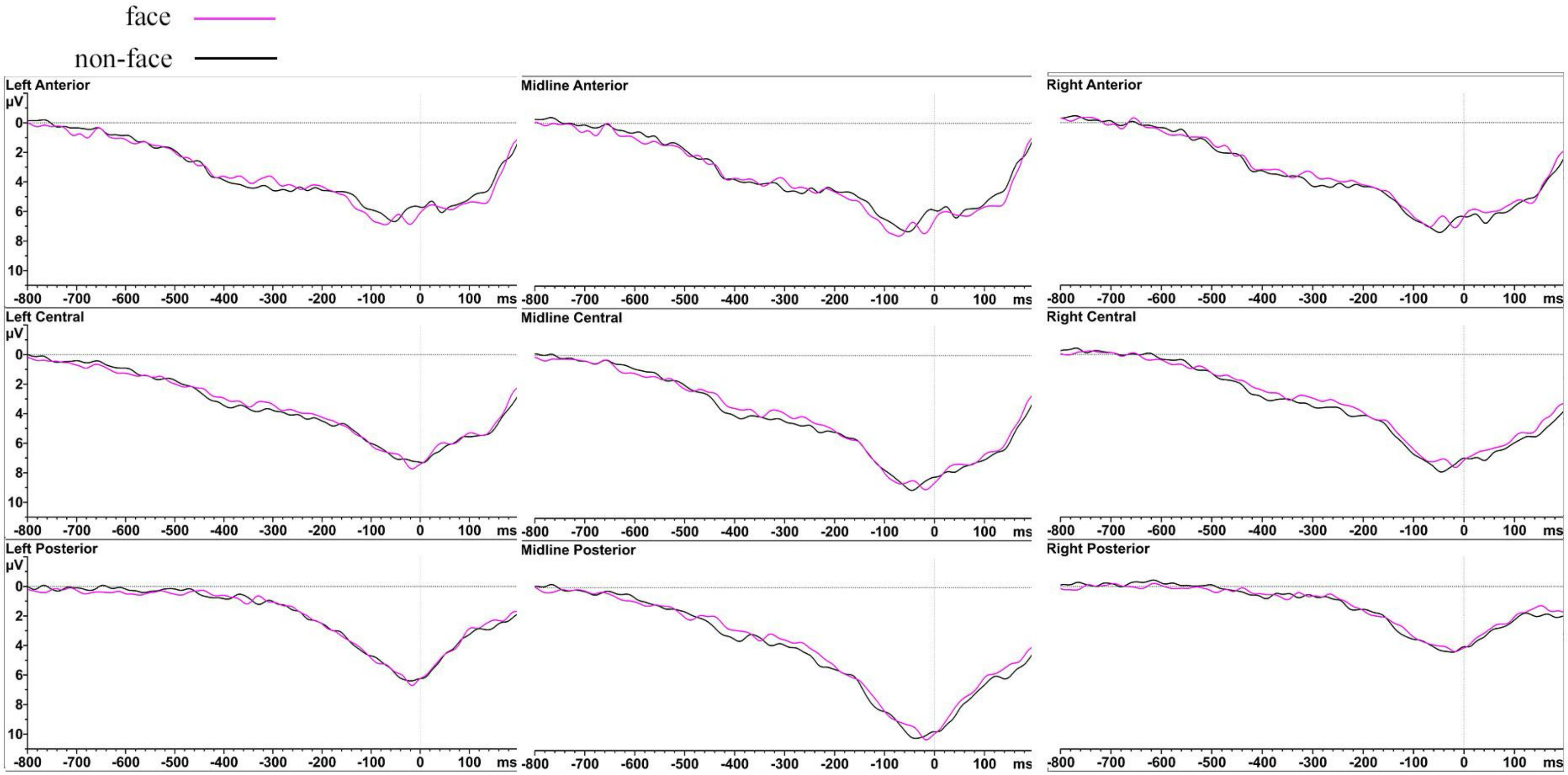

Supplement: Supplementary file 1 — Supplementary Material 1 [file 41598_2025_34117_MOESM1_ESM.pdf]
